# Origin of Co-Expression Patterns in *E.coli* and *S.cerevisiae* Emerging from Reverse Engineering Algorithms

**DOI:** 10.1371/journal.pone.0002981

**Published:** 2008-08-20

**Authors:** Mattia Zampieri, Nicola Soranzo, Daniele Bianchini, Claudio Altafini

**Affiliations:** SISSA-ISAS, International School for Advanced Studies, Trieste, Italy; Center for Genomic Regulation, Spain

## Abstract

**Background:**

The concept of reverse engineering a gene network, i.e., of inferring a genome-wide graph of putative gene-gene interactions from compendia of high throughput microarray data has been extensively used in the last few years to deduce/integrate/validate various types of “physical” networks of interactions among genes or gene products.

**Results:**

This paper gives a comprehensive overview of which of these networks emerge significantly when reverse engineering large collections of gene expression data for two model organisms, *E.coli* and *S.cerevisiae*, without any prior information. For the first organism the pattern of co-expression is shown to reflect in fine detail both the operonal structure of the DNA and the regulatory effects exerted by the gene products when co-participating in a protein complex. For the second organism we find that direct transcriptional control (e.g., transcription factor–binding site interactions) has little statistical significance in comparison to the other regulatory mechanisms (such as co-sharing a protein complex, co-localization on a metabolic pathway or compartment), which are however resolved at a lower level of detail than in *E.coli*.

**Conclusion:**

The gene co-expression patterns deduced from compendia of profiling experiments tend to unveil functional categories that are mainly associated to stable bindings rather than transient interactions. The inference power of this systematic analysis is substantially reduced when passing from *E.coli* to *S.cerevisiae*. This extensive analysis provides a way to describe the different complexity between the two organisms and discusses the critical limitations affecting this type of methodologies.

## Introduction

Reverse engineering a gene network means extrapolating a graph of putative gene-gene interactions from high throughput microarray data. Many algorithms have been proposed for this scope in recent years (see [1], [2], [3] for an overview) and many are the (very) different contexts of application: deduce/integrate/validate various types of “physical” networks of interactions between genes or gene products, see e.g. [4], [5], [6], [7], [8], [9], [10], [11], [12], [13], [14].

**Figure 1:**
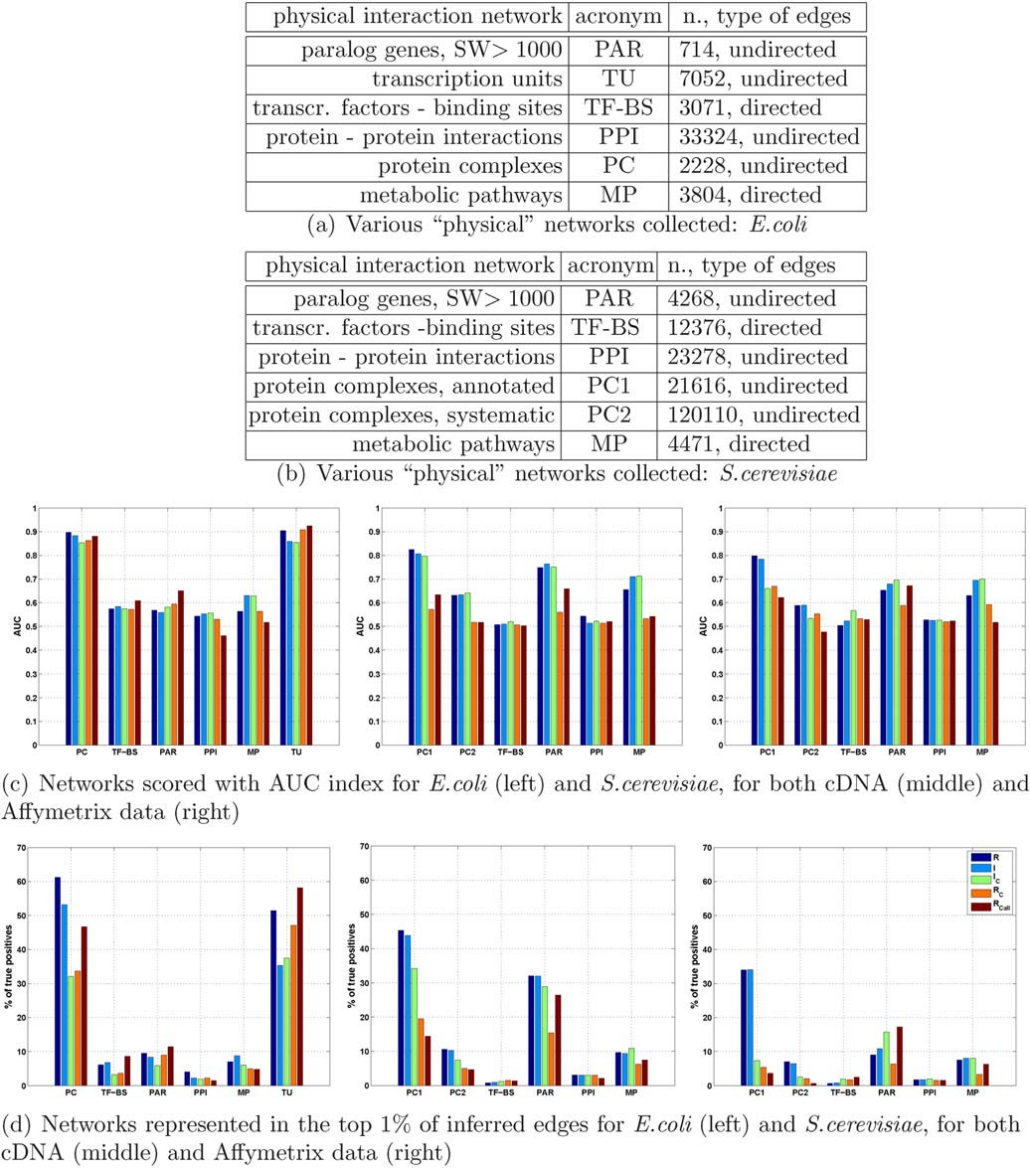
Overrepresented physical networks. For each of the two organisms we collected several networks representing different genomic or physical interaction properties, shown in Table (a) and (b), see Supplementary Notes S1 for data sources. The similarity matrices, computed with Pearson correlation (R), mutual information (I), conditional mutual information (I_c_), partial Pearson correlation (R_c_) and graphical Gaussian model (R_call_) and representing the predicted likelihood of an edge between any two genes, are compared with the graphs of the various networks. The AUC values for the receiving operating characteristic are reported in the histograms for *E.coli* and *S.cerevisiae* (c). In panel (d) a coarse grain statistics is used to describe the results. It consists in sorting the inferred weights, binning them into 100 bins and counting the percentage of “true” edges (of each physical network) lying in each bin. The percentages of true positives in the top bin are shown in the bottom histograms (a randomly chosen network would yield 1% of true positives). The same qualitative conclusions can be drawn from both scoring methods. *E.coli* inference: two networks are neatly emerging, TU and PC. The first emphasizes the visibility in the expression pattern of the operonal structure of the DNA. The TU and PC detected have an overlap which is consistent but still below 50% (of the 2632 TU edges and 1364 PC edges in the top 1%, 694 are in common), meaning that also co-participation in a PC is a strong, independent source of co-expression. *S.cerevisiae* inference (cDNA and Affymetrix data): the dominant index is PC1 in both datasets, followed by the map of duplicated genes. The high magnitude of the peaks in the cDNA data alone strongly suggests that this technology may be affected by a systematic bias towards unspecific binding and cross-hybridization of genes with sequence similarities [46], [16], see also Fig. 6. The intersection of the results for the two platforms basically corresponds to the Affymetrix edges, see Supplementary Notes S6. With the exception of TF-BS for *S.cerevisiae*, all histograms in panel (c) and (d) are statistically significant (q.value <0.05, see Supplementary Notes S1 and S3).

Our aim in this paper is to address the following question: which one among these different networks is more likely to emerge from a completely unsupervised reverse engineering processing of the gene expression data, and at which level of detail can we confidently reconstruct such networks on two model organisms (*E.coli* and *S.cerevisiae*) of different complexity? In other words: what is the most likely biological origin of the pattern of gene-gene expression similarities we see probing only the “layer” of transcripts without adding any *a priori* information neither on the “upstream” regulatory interactions (like a direct transcriptional activation could be considered) nor in the “downstream” ones (at the level of protein or of metabolic interactions)? And finally, how is the organism complexity influencing our ability to retrieve gene-gene interactions via gene co-expression? For these purposes, we choose two model organisms for which large compendia of gene expression microarrays are available and also several networks can be collected from the literature, like maps of transcription factors–binding sites (TF-BS), protein–protein interactions (PPI), protein complexes (PC), and metabolic pathways (MP). In order to take into account also the homology and the architecture of the genomes, we considered maps of paralog genes (PAR) [15] and, for *E.coli* alone, a map of transcription units (TU) describing the operonal structure of the prokaryotic DNA (see Tables (a) and (b) of Fig. 1 and Supplementary Notes S1 for details and data sources). As for gene profiling, we used three different compendia: one for *E.coli* and two for *S.cerevisiae* (one containing cDNA experiments, the other Affymetrix experiments).

For this last organism, as a byproduct, the comparison of the two datasets allows the evaluation of the differences between the two gene profiling technologies (see in particular Fig. 1).

These datasets contain profiling experiments performed in widely different conditions. In the philosophy of reverse engineering [1], [2], [3] this is meant to capture as much as possible of the different perturbations that can be applied to a system. Needles to say reverse engineering algorithms are strongly dependent on the quality and numerosity of the dataset used. In an effort to overcome the limitations of current reverse engineering algorithms and possible biases due to the microarray platform considered [16], in this paper we consider simultaneously five different algorithms and rely on datasets from two different platforms (cDNA and Affymetrix technologies).

Several are the examples of how to conjugate gene expression with one of the cited physical networks, like [6] and [11] where expression similarity (together with sequence compatibility) is used to infer new putative TF-BS edges. Rather than TF-BS, the same comparison between expression similarity and a given network graph can alternatively lead to putative new PPI edges [17], [10], [18]. As a matter of fact, according to [13], for *S.cerevisiae*, gene expression correlation is the most significant among the 17 indexes considered for this scope (including, among others, ontological information, sequence similarity, protein localization and domain structure, etc.). Similar uses of gene expression have been published in the context of metabolic pathways: see e.g. [9], [19], or to predict prokaryotic operonal structure [7], [20]. Needless to say, the integration of several of the “physical” maps above is one of the very often used approaches in the literature [21], [22], [12], [23], [24]. In addition, several studies investigate evolution through the comparison of these physical networks, in particular at the level of transcription circuits [25], [26], [27], [28].

There are several motivations that justify the simultaneous use of gene expression in these and other biological contexts, the first and foremost being that genes, gene products and metabolites form a unique complex interlinked system, whose unraveling is far from complete, especially for what concerns its context-dependence (condition-specific activation of regulatory mechanisms, dynamic behavior, dependencies from internal and external parameters such as nutrients and stimuli, etc.). Another reason is that the gene expression “layer” is the only one that can be measured in such a systematic way. A third reason is that even zooming to this layer alone, the current amount, quality and significance of microarray data is drastically insufficient.

The main task of this paper is to test which, among the physical networks mentioned above, are more represented in the inferred gene-gene networks.

The results show in both organisms that the regulation deriving from the co-participation in the same protein-complex is strongly overrepresented in the pattern of high co-expression. This is observed especially in *S.cerevisiae* where an operonal structure is missing. As the functional category that emerges more significantly for both organisms is co-participation in a protein complex, by suitably clustering the inferred networks the genes can be grouped and the groups matched with the known protein complexes. When we compare the outcome of this cluster matching procedure, we see that the degree of the reconstruction resolution is higher in *E.coli* than in *S.cerevisiae*. Most edges of each PC are correctly inferred and the matching cluster-PC is essentially monogamic.

## Results

### Overrepresented networks comparison

Assuming no prior knowledge, a network structure can be inferred solely from microarray data by means of a genome-wide “similarity matrix” [29] (see Supplementary Notes S1 for definitions and algorithms) and used to test which of the types of interactions listed in Fig. 1 emerge significantly. We carry out two different tests to evaluate the performances of the algorithms. In the former the area under the receiving operating curve (AUC) is evaluated for each metric and network, see Fig. 1 (c), while in the second the edge weights resulting from the statistical analysis are rank-ordered and the percentages of “true” edges of each physical network in the top 1% of the inferred edges are shown in the histograms of Fig. 1 (d). The AUC histograms score the reconstruction of the physical networks without choosing any cutoff on edge weight (a value of 0.5 means that the result is not statistically significant), while with the second test we look for networks for which most of the information is retained in the highest 1% of edges (Supplementary Notes S2 and S3). The conclusions that can be drawn from the two procedures are largely in agreement (and in agreement with Precision/Recall curves, see Supplementary Notes S4 and S5). In particular for *E.coli* (Fig. 1(c)) we observe that an AUC index of 0.9 is reached for the TU map, meaning that the pattern of expression similarity is strongly influenced by the operonal structure of the DNA, as is well-known [7], [20]. The other emerging network, the (manually curated) protein complexes, is relevant also for *S.cerevisiae*. Notice how in *S.cerevisiae* the performances decrease drastically passing from the manually curated protein complexes (PC1) to the complexes identified by means of systematic screening (PC2). This consideration extends to PPI on both organisms: the protein-protein bindings detected by high throughput essays need not correspond to stable bindings and hence to highly correlated patterns of expression. On both organisms the direct transcriptional regulation due to the transcription factors (TF-BS map) is far from being the most relevant indicator. However, while for *E.coli* it remains in the range of significance of other networks (around 6–8% in the most significant bin, like MP), in *S.cerevisiae* the map TF-BS is below the threshold of statistical relevance in both datasets we collected. Concomitant causes such as combinatorial regulatory effects [4] or condition-specific activation of the TF-BS edges [11], [30] certainly play a role in the loss of relevance of this class of interactions. Notice that there is a substantial intersection between the true edges detected from the cDNA and Affymetrix datasets (Supplementary Notes S6), meaning that co-expression among certain genes emerge robustly regardless of the particular type of perturbation applied.

To guarantee an unbiased overall picture of the major differences between the two organisms that emerge when reverse engineering large collections of gene expression profiles, we must ensure that the datasets contain a comparable amount of information in terms of perturbative stimulations on the system. For this purpose on each of the three datasets suitably normalized we compute a gene expression variability index (see Materials and Methods). If on the one hand in *S.cerevisiae* the cDNA dataset shows a higher variability with respect to the Affymetrix dataset (a possible reason for the better inference performances on the former, see Fig. 1 and Supplementary Notes S6), on the other hand the two Affymetrix datasets (one for *E.coli* and one for *S.cerevisiae*) are characterizable by a similar content of variability, see Supplementary Notes S7. This consideration reinforces the claim that the worse results obtained for *S.cerevisiae* are not due to lower quality datasets with respect to *E.coli*, but are likely to reflect a more complex transcriptional regulation [31].

### Clustering: *E.coli*

**Figure 2:**
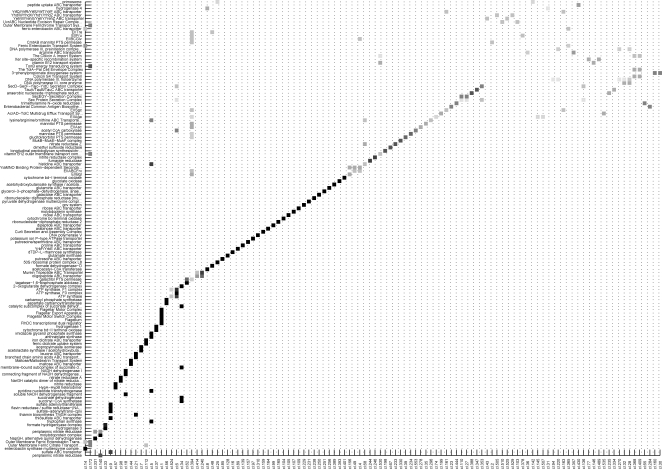
Correspondence between expression clusters and protein complexes for *E.coli* *.* Selecting an acceptance threshold of 0.8 on the Pearson correlation coefficients, we obtain a graph of 19238 arcs involving 1998 genes. This graph is decomposed into 556 clusters (using a hierarchical algorithm, see Methods and Supplementary Notes S10). Of the 556 expression clusters, 114 intersect with 135 protein complexes (having at least 2 genes in the set of 1998 genes passing the correlation threshold, out of the 209 PC). The gray scale indicates the percentage of genes of the PC in the cluster (black is 100 %). The correspondence clusters-PC is almost monogamous (the majority of PC, more than 80, belongs to a single cluster, while more than 120 of the 135 PC are confined to at most 2 clusters, see Supplementary Notes S8 for a more detailed statistical analysis and Supplementary Notes S9 and S11 for the correspondence between clusters and TU).

If we want any clustering algorithm to be effective, the graphs of interactions have to be sufficiently sparse. We adopt this criterion to select a suitable cut-off on the edges weight (see Material and Methods for further details). The edges of highest significance, suitably clustered, can be tested against the most relevant physical networks emerging from the previous analysis. For *E.coli*, the clustered expression correlations reproduce faithfully a large part of the collection of PC, and the matching clusters-PC is quasi-monogamous (see Fig. 2 and Supplementary Notes S8 for details and statistics). A similar (even better) unambiguous correspondence is detected between the clusters and the TU (see Supplementary Notes S9), while for MP the percentages are lower but still significant. Most often co-clustered genes share similar functional annotation and can be used to infer/confirm biological hypothesis.

A thorough description of the ontological information deduced from the cluster analysis is provided in the Supplementary Notes S1. The most striking example is represented by the largest cluster, which includes (in 61 genes) basically all the 50 genes known to be involved in flagellar formation and function. Apart from the flagellum complex subunits (24) and its transcriptional regulators (flhDC and the factor fliA), the cluster contains chemotactic genes, genes regulated by the flhDC complex, by the factor or the anti- factor, other genes involved in flagellar biogenesis and motility, or predicted regulators of the factor. Such a functional compactness (and disconnection from the rest of the gene network, see Supplementary Notes S10) probably originates from *E.coli*'s need to activate the flagellum in every kind of experimental condition and in constant stoichiometric ratio. Also ribosomal genes tend to form large clusters of functionally similar genes (mainly concentrated in clusters 10, 20 and 25) going beyond the operonal structure and forming different ribosomal structural components (rpl, rps, rpm, rpo). Another remarkably homogeneous set of genes not induced by any operon is in cluster 24: of its 10 genes, 9 are associated with the SOS pathway.

The list of significant clusters is long, as essentially all basic functions needed for survival and growth are captured by the cluster analysis. Nucleotide (cluster 56 for pyrimidine, cl. 88 for purine) and amino acid biosynthesis are recurrent biological functions retrieved by the procedure. For this last function, the resolution is often at the level of the single amino acid, like serine biosynthesis and threonine biosynthesis from homoserine (cl. 7), tryptophan and histidine biosynthesis (cl. 5), arginine biosynthesis (cl. 36), methionine biosynthesis (cl. 69, 7), alanine biosynthesis (cl. 404), isoleucine biosynthesis from threonine (cl. 72) and cysteine biosynthesis (cl. 9). The single resolution extends to tRNAs: valine tRNAs (cl. 171), glutamate tRNA (cl. 175), asparagine tRNA (cl. 102), methionine tRNA (cl. 166), glycine tRNA (cl. 167), leucine tRNA (cl. 168), although sometimes similar enzymatic functions prevail (like in cluster 41 where genes involved in amino acid-tRNA synthetase for five different amino acids are grouped).

Biosynthetic pathways are visible for many (other) compounds, like, for example, thiamine (cl. 21), enterobactine (cl. 14), spermidine (cl. 133), etc. Likewise for degradatory pathways (e.g. alanine in cl. 404, threonine in cl. 185, L-arabidose in cl. 26, etc.), and for many elements of the superfamily of ABC transporters.

Responses to various stresses are well detected, like osmotic (cl. 80, 139), oxidative (cl. 415), thermal (cl. 106, 184), acid (cl. 308) and extracytoplasmatic (cl. 340). Also metabolic functions, like for example aerobic and anaerobic respiration, are well identified by specific and disjoint clusters. For instance for the aerobic respiration, cluster 34 contains the sdhCDAB-sucABCD operon involved in the two consecutive succinate-related steps of the TCA Cycle. A cluster related to anaerobic respiration is cluster 117, which contains part of the fixABCX TU, thought to be involved in the anaerobic metabolism of carnitine. This last hypothesis is reinforced by the co-clustering with caiD, a gene having a carnitine racemase activity. Cluster 203 is also significant, containing 3 genes belonging to three different TU but all involved in the anaerobic respiration. The preferred electron acceptor for anaerobic respiration in *E.coli* is nitrate that is reduced to nitrite which is either excreted or further reduced. *E.coli* contains 3 nitrate reductases: two of them, nitrate reductase A (NRA) and nitrate reductase Z (NRZ), are membrane bound, while the third one, Nap, is located in the periplasm. Their different environmental conditions for activation are reflected in the formation of three separate and neatly defined clusters (cl. 98, 233, 140). Similar considerations extend to the 2 nitrite reductases (cl. 57 and 246). In addition, nitrate serves as a nitrogen source, an important constituent of protein and amino acids, and nitrogen metabolism is a function that emerges compactly from our analysis (cl. 3). Iron transport is usually involved in the formation of proteins belonging to the respiration chain, as it has an electron acceptor activity, and is represented here by cluster 19. Assimilation of other substrates such as sulfur and carbon are depicted respectively by clusters 9, 19, 347, and 46, 291, 393.

Several other clusters contain clues about putative gene functions, like cluster 67 encoding for two components of the dmsABC, dimethyl sulfoxide (DMSO) reductase, a terminal electron transfer enzyme functioning anaerobically in absence of nitrate. The other genes in the cluster are paralogs, like, ynfF and ynfE (highly similar to dmsA), ynfG (highly similar to dmsB), and ydfZ. Little is known about ydfZ, but the working hypothesis [32] is that it is activated under anaerobic growth, and the clustering procedure reinforces this assumption. Another example of biological inference is cluster 161. It contains sgcABC, part of the sugar transporting phosphotransferase system (PTS), together with ytfT, that, although part of a different TU, according to sequence similarity may function as an ATP-dependent sugar transporter, hypothesis consistent with our results.

### Clustering: *S.cerevisiae*

**Figure 3:**
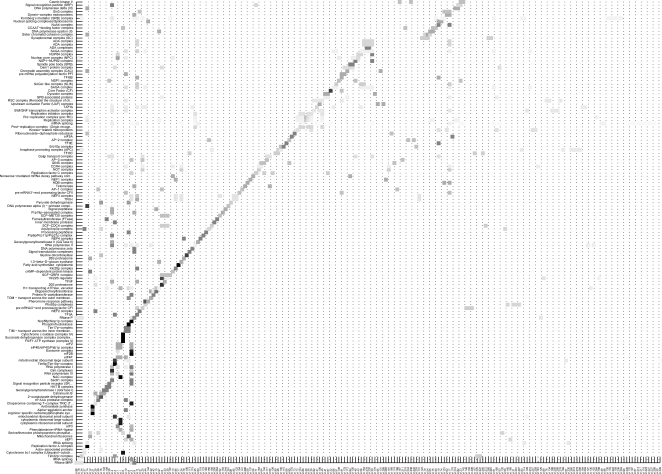
Correspondence between expression clusters and protein complexes (PC1) for *S.cerevisiae* *.* A graph of 1301 nodes and 131679 edges in the intersection of the cDNA and Affymetrix correlation matrices is retained for the clustering. Of the 299 expression clusters obtained, 212 intersect with 141 of the 217 protein complexes drawn from PC1. The gray scale indicates the percentage of genes of the complexes in the cluster (black is 100 %). While the clustering is still sufficiently accurate, the most significant difference with respect to Fig. 2 is the percentage of complex subunits detected in average by the thresholding, implying that the complexes have a lower degree of cohesion in terms of gene expression. A few statistical parameters are provided in Supplementary Notes S12 and S13.

The clustering procedure is repeated also for *S.cerevisiae*, this time merging the two datasets and choosing a lower threshold in order to make an unbiased comparison with previous results for *E.coli* (similar number of edges, see Supplementary Notes S1 for details). As can be seen in Fig. 3, while the correspondence clusters-complexes (of type PC1) is still acceptable, the percentages of subunits detected for the complexes are drastically reduced with respect to *E.coli*. Also qualitatively, the inferred results are quite different, with a few very accurate reconstructions of large complexes but much less information content in the medium-small size clusters. Large and small ribosomal subunits are captured very precisely for both cytoplasmic (cl. 1) and mitochondrial (cl. 3) ribosomes, in agreement with the previous results for *E.coli*. The latter cluster (of 70 genes) is a good example of compartmental homogeneity: the 56 mitochondrial ribosomal genes are in fact co-clustered with 6 more genes from the mitochondrial membrane translocases. Even more compact clusters (in terms of both localization and function) are cluster 6, with 25 of the 32 subunits of the proteasome (out of 34 genes of the cluster), and cluster 5, which contains all the respiratory chain complexes (34 out of 36 genes of the cluster). Notice how in this last case also the main transcriptional regulator of the oxidative phosphorylation (HAP4) is co-clustered, one of the very few examples of TF-BS edges detected. In general, the large clusters tend to co-localize but also to share complex subunits (see the example of the RNA polymerases complexes scattered in clusters 2, 4, and 7). As for the remaining medium-small size clusters, most of those having a significant annotation tend to be involved in transcription and translation processes, while metabolic functions are fragmentary and do not emerge from the clusters, mostly because many enzymatic genes are missing (they have no significant correlation coefficients). For example two pairs of enzymes of glycolysis are co-clustered in cluster 8, but most of the other genes in the pathway are not passing the correlation filter. A few clusters containing eminently metabolic genes are however present (e.g. cl. 12, 15, 21, 30, 31, 100), although they are not pathway-specific. Sometimes genes co-localize also in other compartments like the endoplasmic reticulum (15), the cytoskeleton (37) or the Golgi vesicles (117).

An example of how to use the clustering in the verification of uncertain functional annotations is the following. The gene PPE1 (YHR075C, also known as MRPS2) among other annotations, is also identified as a small subunit mitochondrial ribosomal protein [33], [34], an annotation which is contradictory with e.g. the results of [35]. In our analysis PPE1 is lost at the correlation filter, meaning that it has no strong and stable interaction with any other gene. Extending for example to the 10 “newly” reported subunits of mitochondrial ribosomes of [34], 7 are correctly included in cluster 3 and 1 in cluster 8 (still mitochondrial) and only 2 are missing (YMR158W and YPL013C).

### Influence of gene distance

**Figure 4:**
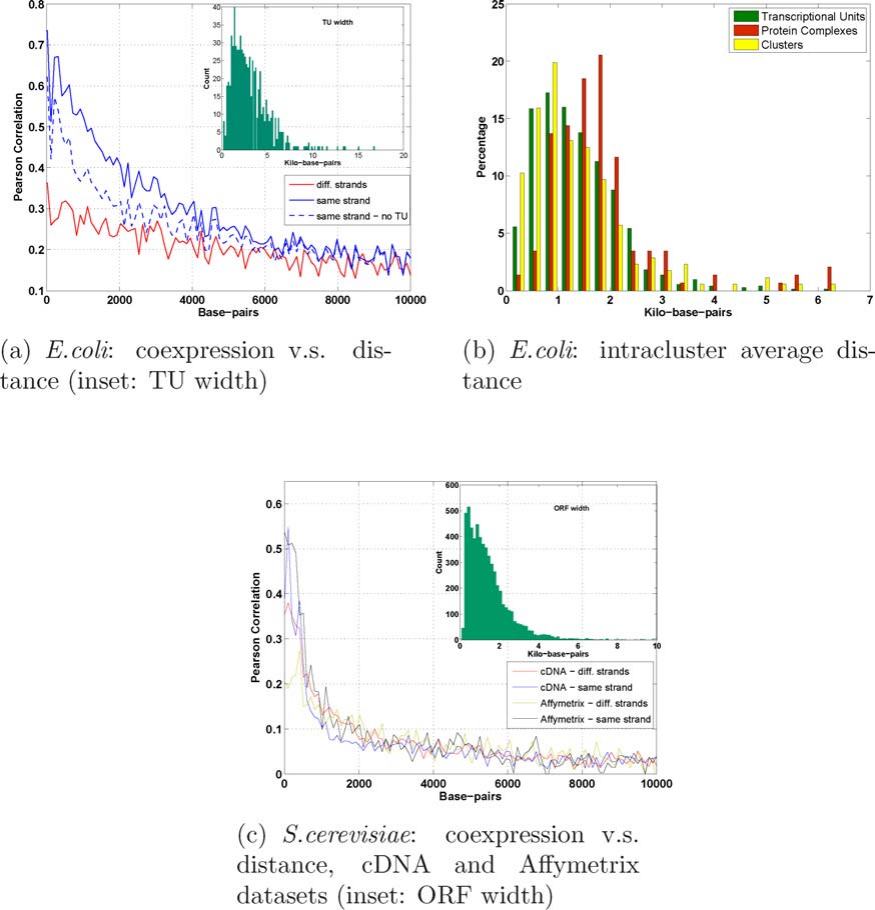
Pearson correlation and distance on the genome. Co-expression decays more rapidly with distance in *S.cerevisiae* than in *E.coli*: the correlation drops to 0.2 at a distance of 6 Kbp in *E.coli* (a), as opposed to 1 Kbp in *S.cerevisiae*, for both cDNA and Affymetrix datasets (c). In *E.coli* the value 6 Kbp is consistent with the distribution of TU width (inset panel in (a)). Genes on the same strand have much higher correlation than genes on opposite strands. For *E.coli*, even if we restrict to gene pairs not involved in a TU (see dashed blu line in (a)), the influence of distance on co-expression is still clearly visible. In *S.cerevisiae*, the short-range high correlation peak is represented almost completely by overlapping ORFs (the distribution of ORF widths is shown in the inset), for which the cDNA experiments cannot discern any strand-specificity, unlike Affymetrix experiments. In panel (b), the distribution of intracluster average distances (see Supplementary Notes S1) for *E.coli* is compared with the corresponding distributions of average distances among PC and TU subunits. The histogram for the clusters is more similar to that of TU than PC, although its tail is heavier and more related to PC. A similar analysis is impossible for *S.cerevisiae* as the vast majority of clusters is composed of genes located on different chromosomes.

**Figure 5:**
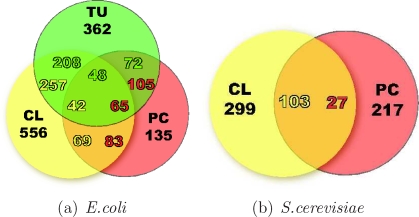
Overlap between the clusters and the main physical networks for *E.coli* and for *S.cerevisiae* *.* The Venn diagram for *E.coli* shows how many groups of genes of one of the three categories, clusters, TU and PC, are completely contained in the groups of the other two (monochromatic inclusion: a group of genes of type X belongs to a single group of type Y, see Fig. 1 for the TU/PC overlap with a more relaxed criterion). For example there are 72 TU contained in the 135 PC, and 105 PC contained in the TU. Of these 105, 65 are completely included simultaneously in TU and clusters (for the metabolic pathways see Supplementary Notes S14 and S15). For what concerns the ability of the clustering to infer PC and TU, if in absolute terms the correspondence clusters/TU is certainly higher, in percentage it is of the same order (61% for PC and 57% for TU). These percentages are much higher than in *S.cerevisiae* (10%), see (b), as can be deduced visually comparing Fig. 2 and Fig. 3.

For *E.coli*, the operonal structure of the genome is certainly a key factor in the formation of the clusters [20], [7]. In Fig. 4 (a) and (c), co-expression of genes located adjacent to each other on the genome is quantified and genes belonging to the same or to different strands are distinguished. However, the operonal structure alone does not exhaust the information that can be extrapolated from the expression correlation patterns (see Fig. 4 and Fig. 5). We can notice for instance that the distribution of intracluster average gene distances (shown in Fig. 4(b)) although largely comparable to that of the TU, has a heavier tail, more related to the PC distribution. Most of the large clusters are examples of functional information not exhausted by any operonal structure. It is interesting to notice that the difference in the overlap clusters/TU concerns most often the genes located at the boundaries of the operons (see e.g. cl. 3, 5, 6, 10, and many more). In spite of this, as a confirmation that the operonal structure and/or protein complex interactions are much stronger mediators of co-expression than direct DNA binding (i.e. being a pair of TF-BS), we notice that co-clustering of these last pairs are sporadic (e.g. cl. 1, 3, 7, 24, 38, 74, 101). The influence of the genes distance on their co-expression is noticeable to some extent also in *S.cerevisiae* [36] but decays more rapidly than in *E.coli* (see Fig. 4(c)). While the decay/distance ratio is similar on the cDNA and Affymetrix datasets, for contiguous genes the former is unable to distinguish strain specific genes.

## Discussion

The systematic observation of the patterns of gene co-expression, inferred from compendia of experiments, tends to unveil functional categories that are stable (i.e. co-participation in a complex, co-localization, similar biological function, etc.) rather than transient or condition-specific (i.e. TF-BS) [47]. The picture emerging from the genome-wide analysis shows common aspects in the two organisms, like the co-existence of various “layers” of regulation, or the importance of the physical interactions among the gene products in determining co-regulated expression patterns. Many observations are hints of the different complexity characterizing the two model organisms. One such result is a marked decrease into the statistical significance of the direct transcriptional control when passing from the prokaryotic to the eukaryotic genome. The increase in the complexity of regulatory mechanisms, genome architecture and number of functions per gene can be the main reason for our inversely proportional ability to retrieve significant and detailed information by means of a reverse engineering approach. This suggests that reverse engineering methods should be used with care for higher organisms for which the prediction of interactions from gene expression is often considered an ill-posed problem [48].

## Materials and Methods

### Gene expression databases and assessment of the perturbational content

We downloaded the “Many Microbe Microarrays Database” (from http://m3d.bu.edu, T. Gardner Lab, Boston University [37]) for *E.coli* (445 experiments for 4345 genes) and compiled two separate collections of microarrays for *S.cerevisiae*, one containing experiments performed with cDNA chips (958 experiments for 6203 ORF) the other with Affymetrix platform (790 experiments, all performed with the GeneChip Yeast Genome S98 platform and all downloaded from Gene Expression Omnibus, http://www.ncbi.nlm.nih.gov/geo/). All 3 datasets were normalized prior to network inference. In order to compensate for platform-specific or organism-specific absolute expression abundances, a quantile normalization is applied. This yields an identical distribution to all experiments of each dataset. The perturbational content of a normalized dataset is computed by means of a gene expression variability index equal for each gene to the percentage of experiments in which gene expression is an outlier with respect to a confidence interval centered on the mean value and of width equal to twice the standard deviation. Repeating the calculation of this expression variability index on subsets of experiments of different sizes yields coherent results, see Supplementary Notes S7.

### Physical networks

The various networks collected are listed in Table 1(a) and (b) of Fig. 1 of the paper. The information about duplicated genes is downloaded from the SSDB database of KEGG (http://www.genome.jp/kegg/ssdb/). Networks of paralog genes (PAR) are constructed computing pairwise similarities by means of the Smith-Waterman (SW) algorithm with acceptance threshold fixed to 1000 (100 is the default minimum set by KEGG). We obtained TF-BS networks from the *RegulonDB* database (http://regulondb.ccg.unam.mx), version 5.6, for *E.coli* [38], and from a recent collection [4] for *S.cerevisiae*. For *S.cerevisiae*, PPI and protein complexes networks were downloaded from the MPACT subsection of the CYGD database at MIPS (http://mips.gsf.de/genre/proj/mpact/). The complexes annotated from the literature and those obtained from high throughput experiments (according to the MIPS classification scheme these last are labeled “550”) were kept separated and denoted respectively PC1 and PC2. Since the corresponding PPI information from SGD (http://www.yeastgenome.org/) and DIP (http://dip.doe-mbi.ucla.edu/) databases overlap for more than 50% with the MIPS PPI and PC, these will not be considered further for the analysis. Tables of Transcription units (TU) and PC for *E.coli* were downloaded from RegulonDB and EcoCyc (http://ecocyc.org/), and high throughput PPI data from recent studies [39], [40]. The PPI network contains as a subset the DIP database. The metabolic pathways (MP) are compiled from the tables of biochemical reactions developed by Palsson group (see http://gcrg.ucsd.edu/In_Silico_Organisms). Reference publication for *E.coli* MP is [41] and for *S.cerevisiae* MP [42]. Nodes of these MP networks are enzymatic genes, and a direct edge exists between two nodes when a product of the reaction catalyzed by one gene is a substrate of the reaction catalyzed by the second gene. The MP networks considered here are the enzyme projections of the reaction graphs. To avoid overdense graphs, isoenzymes and common abundant reactants like CO2, ATP, ADP, GLU, NAD, NADH, NADP, NADPH, NH3, PI, PPI were neglected.

### Similarity measures

We used Pearson correlation (*R*), mutual information (*I*), conditional mutual information (*I_c_*), partial Pearson correlation (*R_c_*) and graphical Gaussian model (R*_call_*) as similarity measures. While correlation-based measures are linear, entropy-based measures like the mutual information have a nonlinear nature. See Supplementary Notes S1 for details.

### Overrepresented networks

In the statistical analysis shown in Fig. 1, AUC is the area under the receiving operating characteristic curve [43]. Overrepresentation is detected with respect to a uniform distribution of true edges in the graph, and the level of significance of each top bin in each network is assessed by means of a permutation test with multiplicity correction (see Supplementary Notes S1).

### Clustering procedure

For both organisms, only the Pearson correlation is used for the clustering (the mutual information gives results which are quantitatively very similar). In order for a clustering procedure to be effective, sparser graphs that the previously used 1% of edges must be considered. Once the acceptance threshold on the correlation coefficients is chosen (see below), the graph whose edges pass the correlation threshold is first decomposed into disconnected components. For both organisms, a single connected component turns out to be much larger than the remaining disjoint subgraphs. This large component is therefore decomposed further using a hierarchical clustering algorithm, with weighted average linkage as cost of merging, and taking as number of clusters the number of cuts of size 1 (i.e., of bipartite partitions of the graph joined by a single edge). In the choice of the correlation threshold, there is a trade-off between coverage (i.e., number of nodes with at least an edge above the cut-off, call it ν), and the connectivity degree of the nodes (representing the density of edges in the “surviving” graph). If μ is the number of disconnected components and η the final number of clusters (total of the number of clusters in which the large connected component is subdivided plus the μ−1 other disconnected components), then η/μ is a (approximate) measure of the connectivity growth ratio (η/μ≥1) and ν/*n* of the coverage ratio (0<ν/*n*≤1). The trade-off between the two can be measured for example by the logarithmic sum
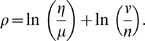
(1)


The thresholds on the correlation coefficients for the two organisms are chosen so as to yield a similar value for ρ. After this clustering procedure, a row/column permutation algorithm based on the Dulmage-Mendelsohn decomposition [44] is applied to “diagonalize” the matrix of correspondences between the cluster and the physical network under consideration (further details in Supplementary Notes S1).

### Semantic similarity

**Figure 6:**
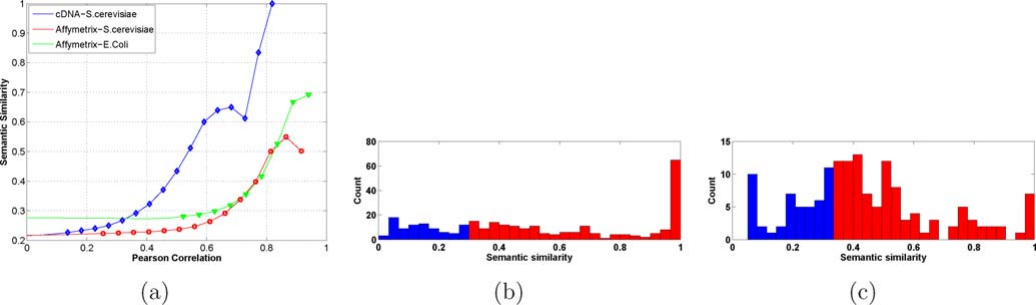
Pearson correlation and semantic similarity. We used a quantitative measure of semantic similarity between gene products (see Supplementary Notes S1) in order to evaluate whether genes with similar function share similar expression profiles. When comparing semantic similarity with co-expression, (a), we see that rather than organism-specific, the differences are platform-specific. If for Affymetrix data the two graphs are similar, the curve grows much faster for cDNA data. This seems to be due to the more unspecific hybridization that characterizes cDNA chips: since genes are often annotated according to sequence similarity, the cross-hybridization bias is amplified towards highly co-regulated pairs [46]. The peak in correspondence of the maximal intracluster semantic similarity in *E.coli*, (b), reflects the matching clusters/operons and is missing in *S.cerevisiae*, where however a sufficiently high degree of functional homogeneity still characterizes the majority of the clusters (bins in red have p.value ≤0.05, see Supplementary Notes S1).

The semantic similarity measure of Fig. 6 is drawn from [45], and the associated p.value by means of a bootstrapping method, see again the Supplementary Notes S1 for full detail.

### Genes physical distance

Each gene in *E.coli* is annotated with starting and ending positions and with strand information (+ or −); in *S.cerevisiae* also the chromosomes are taken into account. Using this information a matrix of pairwise distances was calculated both for *E.coli* and *S.cerevisiae*. Each gene is positioned in the middle of its start and end coordinates. In *S.cerevisiae* the distance was considered only for genes on the same chromosome. In Fig. 4 of the paper, the intracluster average distance is computed as the mean over all pairwise distances among the genes of a cluster. The same measure is computed also for TU and PC. Clearly for each TU this average distance is strictly less than the TU width (shown in the inset of Fig. 4(a) of the paper). In *S.cerevisiae* the population of clusters whose genes co-localize on the same chromosome is statistically too small to give a significant distribution.

## Supporting Information

Supplementary Notes S1 (0.24 MB PDF)

Supplementary Notes S2 (0.07 MB PDF)

Supplementary Notes S3 (0.11 MB PDF)

Supplementary Notes S4 (0.11 MB PDF)

Supplementary Notes S5 (0.24 MB PDF)

Supplementary Notes S6 (0.04 MB PDF)

Supplementary Notes S7 (0.05 MB PDF)

Supplementary Notes S8 (0.04 MB PDF)

Supplementary Notes S9 (0.06 MB PDF)

Supplementary Notes S10 (1.80 MB PDF)

Supplementary Notes S11 (0.04 MB PDF)

Supplementary Notes S12 (0.04 MB PDF)

Supplementary Notes S13 (14.39 MB PDF)

Supplementary Notes S14 (0.04 MB PDF)

Supplementary Notes S15 (0.04 MB PDF)
