# Supplementary material for: Origin of Co-Expression Patterns in E.coli and S.cerevisiae Emerging from Reverse Engineering Algorithms
Source: PLoS One. 2008 Aug 20;3(8):e2981. doi: 10.1371/journal.pone.0002981 (PMC2500178; doi:10.1371/journal.pone.0002981)
Supplement: Supplementary Notes S1 — (0.24 MB PDF) [file pone.0002981.s001.pdf]

SUPPLEMENTARY NOTES
for
Origin of co-expression patterns in *E.coli* and *S.cerevisiae* emerging from reverse engineering algorithms

M. Zampieri, N. Soranzo, D. Bianchini and C. Altafini
SISSA-ISAS, International School for Advanced Studies
via Beirut 2-4, 34014 Trieste, Italy

July 26, 2008

Contents

1 Methods and data **1**
1.1 Data collected . . . 1
1.2 Algorithms . . . 1
1.3 Overrepresented networks . . . 3
1.3.1 Statistical analysis . . . 3
1.4 Clustering . . . 3
1.5 Semantic similarity . . . 4
1.5.1 Statistical analysis . . . 5

2 *E.coli* correlation clusters **6**
2.1 Functional annotation of the clusters . . . 6

3 *S.cerevisiae* correlation clusters **33**
3.1 Functional annotation of the clusters . . . 33

1 Methods and data

1.1 Data collected

The quality of the information gathered in Table 1(a) and Table 1(b) of the paper is of course very diverse. For example the network of *E.coli* TF-BS is highly accurate and constantly up to date with the literature. On the contrary, the PPI maps, compiled mostly from yeast-two-hybrid and TAP mass high throughput experiments, suffer from a very high rate of false positives and are far from complete. The same is true also for metabolic networks. The overlap between the various physical networks is very low (see Table S1 and Table S2), meaning that these networks tend to coexist in an organism, as expected.

1.2 Algorithms

The five similarity measures used are Pearson correlation and mutual information, first order partial Pearson correlation [1], "full" order partial correlation as defined in the theory of graphical gaussian models [7] and conditional mutual information. The different concepts are only partially redundant, see [8] for details. For

Table S1: Intersections between the physical networks described in Table 1(a) of the paper for *E.coli.* Upper triangular part: intersection between the edges. Lower triangular part: intersection between nodes.

$\cap$	PAR	TU	TF-BS	PPI	PC	MP
PAR		12	2	46	4	19
TU	235		80	230	1014	69
TF-BS	11	49		22	3	0
PPI	319	1727	143		158	36
PC	88	419	8	392		25
MP	59	235	0	389	55	

Table S2: Intersections between the physical networks described in Table 1(b) of the paper for *S.cerevisiae.* Upper triangular part: intersection between the edges. Lower triangular part: intersection between nodes.

$\cap$	PAR	TF-BS	PPI	PC1	PC2	MP
PAR		2	176	218	272	152
TF-BS	925		23	9	26	0
PPI	849	3078		1601	1863	22
PC1	197	832	992		5248	6
PC2	472	1595	1866	754		59
MP	240	456	385	37	215	

example, correlation-based measures are linear and assume an underlying gaussian probability distribution of the marginals, while entropy-based measures like the mutual information have a nonlinear nature. Likewise, conditional similarity measures are more prone to describe causal dependencies in the data, while non-conditioned measures are instead "static".

Let m be the number of experiments available and n the number of genes. Assume X_i and X_j, $i, j = 1, \ldots, n$, are random variables representing the genes, and $x_i(\ell)$, $x_j(\ell)$, $\ell = 1, \ldots, m$, their sample measurements. The matrices of edges weights are computed using the following five algorithms, see [8] and references therein for details:

- Pearson correlation:
$$R(X_i, X_j) = \left| \frac{E[(x_i - \bar{x}_i)(x_j - \bar{x}_j)]}{\sqrt{v_i v_j}} \right|, \qquad (1)$$
where $\bar{x}_i$, v_i and $\bar{x}_j$, v_j are means and variances of x_i and x_j over the m experiments and $E[\cdot]$ denotes expectation.

- Partial Pearson correlation
$$R_{C_1}(X_i, X_j) = \min_{k \neq i,j} \left| \frac{R(x_i, x_j) - R(x_i, x_k)R(x_j, x_k)}{\sqrt{(1 - R^2(x_i, x_k))(1 - R^2(x_j, x_k))}} \right|. \qquad (2)$$

- Graphical gaussian method
$$R_{C_{\mathrm{all}}}(X_i, X_j) = \left| \frac{\omega_{ij}}{\sqrt{\omega_{ii}\omega_{jj}}} \right|, \qquad (3)$$
where $\Omega = (\omega_{ij})$ is R^{-1} if R^{-1} exists, it is the small-sample estimate of [7] when R is not full-rank. De facto, $R_{C_{all}}$ is computed by means of the R package GeneNet version 1.0.1, available from CRAN (http://cran.r-project.org).

- Mutual information

$$I(X_i; X_j) = \sum_{\phi_i,\, \phi_j \in \mathcal{H}} p(\phi_i, \phi_j) \log \frac{p(\phi_i, \phi_j)}{p(\phi_i)p(\phi_j)}, \tag{4}$$

where $p(\phi_i)$ is the probability mass function $p(\phi_i) = Pr(X_i = \phi_i)$, ϕ_i in the alphabet $\mathcal{H}$, and likewise for the joint probability function $p(\phi_i, \phi_j)$.

- Conditional mutual information

$$I_C(X_i; X_j) = \min_{k \neq i,j} \sum_{\phi_i,\, \phi_j,\, \phi_k \in \mathcal{H}} p(\phi_i, \phi_j, \phi_k) \log \frac{p(\phi_i, \phi_j | \phi_k)}{p(\phi_i|\phi_k)p(\phi_j|\phi_k)}. \tag{5}$$

1.3 Overrepresented networks

In the datasets collected, the ratio m/n is very low for both organisms, meaning that the inference power is also very low (see [8] for considerations on the predictive power for varying m). Apart from (standard) ROC and precision/recall curves, the "coarse grain" statistics are obtained ranking the elements of the $n \times n$ matrices of inferred weights, binning the ranked weights into 100 bins of equal cardinality, and counting the percentage of "true" edges (of each physical network) lying in each bin. Regardless of the number of edges, a random permutation of the true edges in the $n \times n$ matrices corresponds to a uniform distribution of these edges in the bins, i.e., a percentage of 1% of the true edges in each bin. Overrepresentation of the true edges in the rightmost part of the histogram means that the inferred network has some degree of similarity with the physical network considered. In absolute terms, this degree of similarity remains very low, as each bin contains a huge number of edges ($(n^2 - n)/200 = 94373$ for *E.coli* and 192355 for *S.cerevisiae*). However, the criterion is fair if the aim is to compare different "true" networks (see following paragraph for a significance test on each physical network). Using the 5 algorithms (1)-(5), the distributions of true edges for the 6 networks of *E.coli* are shown in Fig. S2, and for *S.cerevisiae* in Fig. S3 for both the cDNA and the Affymetrix datasets. While the two "static" similarity measures, Pearson correlation (1) and Mutual Information (4), give almost identical results in terms of overrepresentation in the most significant bin, the 3 conditional similarity measures are less in agreement and are probably much more sensitive to numerical problems in the "difficult" range $m/n \ll 1$.

1.3.1 Statistical analysis

If the basic statistics shown in Fig. 1 of the paper and recalled above only aim at detecting overrepresentation with respect to a uniform distribution of arcs in a graph, a more refined test can also be set up to asses the level of significance of the true positive edges detected in each bin of each network. For this purpose, we use a permutation test, which allows to define a null statistics for each physical network, and compute the p.value as the probability that the random null statistics exceeds the observed statistics over multiple permutations. Once the distribution of empirical p.values has been generated, a q.value correction for multiple tests is used [9].

1.4 Clustering

For *E.coli*, selecting an acceptance threshold of 0.8 on the correlation coefficients, we obtain a graph of 19238 arcs involving 1998 genes (and $\rho = 0.34$). This graph has 183 disconnected components (shown in Fig.S10(a)). The larger connected component (which alone involves 1403 genes) is further decomposed using the above clustering algorithm. The overall decomposition into 556 clusters is shown in Fig.S10(b). Of these, 432 are composed of 2 or more genes. We computed the overlap between the correlation-based clustering and some of the most relevant physical networks, namely TU, PC and MP. Of the 2684 TU classified in RegulonDB, (719 uniques and involving more than one gene) we obtain 362 that involve 2 or more genes among the 1998 filtered for expression correlation. These 362 TU intersect 365 of the 556 expression clusters described above. Application of the row/column permutation algorithm mentioned above to the TU-cluster

graph reveals a quasi monogamous matching, see Fig. S9, i.e., for the genes surviving the correlation filter the clusters reproduce to a large extent the operonal structure (see statistics in Fig. S11). As for PC, of the 747 PC we downloaded from EcoCyc, 209 involve 2 or more genes. Further filtering PC for the genes that have at least a correlation passing the 0.8 threshold leaves us with 135 PC. These 135 complexes are represented in 114 of the 556 expression clusters, as shown in Fig. 2 of the paper. Again the correspondence PC-expression clusters is extremely high (see statistics in Fig. S8). When we consider the metabolic pathways, instead, the correspondence clusters-MP is slightly lower, but still significant. The 133 EcoCyc pathways having at least 2 enzymatic genes above the correlation threshold intersect 141 clusters. There is still a good monogamic correspondence between clusters and metabolic pathways, although the percentage of genes of each pathway detected by a cluster is normally lower than for TU and PC, see Fig. S14 and Fig. S15 for the statistics.

For *S.cerevisiae*, both the connectivity degree and the coverage of the graph vary very differently with the correlation coefficients with respect to *E.coli.* In order to attain a similar value of ρ in the intersection of the two correlation matrices (cDNA and Affymetrix experiments), the acceptance threshold for the correlation has to be chosen low (0.2). In this way we obtain a graph of 131679 edges among 1301 genes. This graph, which is almost completely connected (see Fig. S13(a)), is clustered according to the procedure described above. Fig. S13(b) shows large green diagonal blocks appearing, corresponding to parts of large protein complexes correctly identified. The number of clusters obtained is 299. We looked at 267 protein complexes from MIPS (only among those manually annotated, i.e., belonging to PC1 in the notation of Table 1(b) of the paper), and retained the 141 complexes composed by at least 2 genes among those 1301 genes that passed the correlation threshold (out of 217 PC1 surviving the thresholding). Of the 299 correlation clusters identified above, 212 have nonempty intersection with these 141 complexes. The matching between clusters and PC is shown in Fig. 3 of the paper. The application of the same procedure to the metabolic pathways of *S.cerevisiae* yields results of negligible significance, as already mentioned in the paper.

1.5 Semantic similarity

The package SemSim, based on the Gene Ontology package GO v1.9, developed for R (www.bioconductor.com), was used to calculate the functional similarity among pairs of genes. The "semantic similarity measure" is based on the notion that the less frequently used terms are more informative. So given the GO DAG, the "information content" is defined as the number of times each term, or any child term, occurs in the structure and it is expressed as a probability (p) ranging from 0 to 1 and increasing monotonically when moving from the leaves toward the root node ($p = 1$). There are 3 semantic similarity measures implemented, and all of them use the information content of the shared parents of the two terms. As GO allows for multiple parents for each concept, two GO terms c_i and c_j can share parents by multiple paths; so the minimum probability associated to the content, $\min_{c=\{c_i,c_j\}} p(c)$, is taken as the information content associated to the term. In particular, of the three possible types of measure for semantic similarity included in the package (Resnik, Lin, and Jiang), the Lin measure for "Biological Process" (BP) terms was chosen [5]. According to [3], the performances of this measure are similar to those of the Resnik measure, but this quantity is more tractable, ranging from 0 (unrelated concepts) to 1 (identical concepts), and making it a more suitable quantity for our purposes. Denoting $S(X_i)$ the set of GO term annotations for the gene X_i, the semantic similarity between genes X_i and X_j is computed as

$$\mathrm{sim}(X_i, X_j) = \frac{2\ln(\min_{c=\{c_i,c_j\}}\{p(c)\})}{\ln p(c_i) + \ln p(c_j)}, \qquad c_i \in S(X_i), \;\; c_j \in S(X_j). \tag{6}$$

To be more precise, if gene products are annotated with multiple GO terms, the maximum GO term similarity is considered, while the annotations associated with "BP unknown" are eliminated. The result is a 2833x2833 matrix for E.Coli, and a 4270x4270 matrix for *S.cerevisiae* of pairwise BP similarities between genes. The next step is to calculate a measure of compactness and distance, respectively for each cluster and each pair of clusters. The first one is determined for those clusters with more than one gene annotated, as the mean of the semantic similarity (6) of all the possible pairs in that cluster. Instead the measure of distance between two clusters is computed as the mean of all possible similarities between the genes included in one cluster

versus the genes included in the other one. The intracluster semantic compactness is shown in Fig. 6 of the paper. The intercluster semantic similarities in the two organisms yield very similar histograms (not shown).

1.5.1 Statistical analysis

In both organisms the mean cardinality of a cluster is approximately 4 genes. The mean value for the functional compactness over thousand of random sets of 4 genes has been computed, for both the organisms, in order to asses a level of significance (empirical p.value) for the intracluster compactness. All the clusters with a compactness p.value below the threshold of 0.05 are considered significant and depicted in red bars in Fig. 6 of the paper.

2 *E.coli* correlation clusters

As the datasets used contain microarray experiments taken in a variety of conditions (genetic perturbations, response to different antibiotics, stress conditions and temperature, variation of PH, substrates, growth phases), it is reasonable to deduce that the functional moduli detected correspond to the most stable ones for *E.coli.* Some of the most interesting clusters are described in the text. Here we provide a detailed description of the resulting annotation cluster by cluster. Apart from those mentioned in the paper, clusters of particular interest are the following: 11, 26, 34, 40, 56, 61, 70, 78, 85, 87, 89, 97, 117, 136, 139, 142, 144, 161/163, 166, 172, 173, 268, 282, 286, 288, 365.

The observation, in Fig. 4(a) of the paper, that neighboring genes tend to be co-regulated also in absence of (known) operonal structure is supported by a wealth of examples, see clusters 17, 29, 35, 54, 55, 71, 78, 91, 93, 118, etc. In the paper it is also claimed that the operonal structure does not exhaust the functional information contained in the clusters. Most of the large clusters are examples of this. For other examples in which the PC structure is prevailing over the TU organization see e.g. clusters 97, 244, 389. For MP prevailing over TU see cl. 36, 56, 88, etc.

For genes involved in multiple processes/compounds the classification is much less effective. An example is the gene ptsH which codes for a subunit of glucitol/sorbitol PTS permease, CmtAB mannitol PTS permease, galactitol PTS permease, mannose PTS permease, mannitol PTS permease, N-acetylglucosamine PTS permease, and at the same time, enters into many enzymes II, such as EIIFru, EIIABCFrv, EIIBCMurP, EIIAsc, EIIBgl, EIIChb, EIIBCGlv , EIIHrsA, EIIBCMalX, EIITre, EIIAga, EIISga, EIIBCFrw and EIISgc. The gene ptsH is clustered with (totally functionally disconnected) gapA (cluster 394).

2.1 Functional annotation of the clusters

Cluster n. 1 (of 61 genes) The largest cluster obtained has 61 genes and contains basically all the 50 genes involved in flagellar formation and function. Following the EcoCyc table of complexes, among these 61 genes are the 24 genes (of class II and III) forming the subunits of the flagellum complex (flagellar motor complex, flagellar export apparatus and flagellar switch complex) and those involved in its transcriptional regulation, the class-I complex flhDC (formed by the genes flhC and flhD) and the σ^{28} factor (fliA). All the components of these complexes belong to the cluster. Notice that the σ^{70} factor genes instead are not included in the cluster. The majority of the remaining genes in the cluster are also strictly related to the flagellum, functionally or transcriptionally: chemotactic genes (tap, tar, trg, tsr, cheA, cheB, cheR, cheW, cheY, cheZ, fliK, fliL), genes regulated by the flhDC complex (flhE, flgA, fliS, fliT, yecR), by the σ^{28} factor (flxA), or anti-σ^{28} factor (also regulated by flhDC: flgM, flgN), other genes involved in flagellar biogenesis and motility (flgD, flgJ, fliL, ycgR), or predicted regulators of σ^{28} factor (fliZ). For the few remaining genes little is known. yecR is known to be regulated by flhDC. FliC, or flagellin, is the basic subunit that polymerizes to form the rigid flagellar filament of *E.coli.* The promoter of ynjH (b1760) is predicted to be σ^{28}-dependent, b1044 seems to be involved in cell adhesion and transcription of ves (b1742, also included). The last three genes in the cluster yedM, yjdA and yjcZ have no annotation. Seen in terms of TU, the cluster contains entirely fglAMN, fglBCDEFGHIJ, fglKL, flhADE, fliC, fliDST, fliE, fliFGHIJK, fliLMNOPQR, flxA, tar-tap-cheRBYZ, motAB-cheAW, trg, tsr, ves, yecR. As for the fliAZY operon, it misses fliY which belongs to cluster 20. For the fliY homolog gene in Salmonella typhimurium, transcription is due to both flagellar and non-flagellar promoters, suggesting that it may not be a flagellar gene [4]. Our clustering reinforces this suggestion.

Cluster n. 2 (of 31 genes) The genes sgbE and sgbU belong to the same TU yiaKLMNO-lyx-sgbHUE and are co-clustered with nearby genes yiaA and yiaB. The TU yghRS is fully included, while yqiI, yqiH and yqiG are part of insC-5D-5-yqiGHI-insCD-5 TU. bglG and bglB belong to bglGFB, idnO and idnD to idnDOTR. Most of the genes are involved in carbohydrate catabolism.

Cluster n. 3 (of 27 genes) It contains the TU csiD-ygaF-gabDTP (except for gabP) and the gabDTP transcriptional regulator nac. The cluster is shared with the rutABCDEFG TU which (like gabDTP) is

involved in nitrogen metabolism. Also the gene of the nitrogen regulatory protein GlnK belongs to this cluster and so does amtB whose product, AmtB ammonium Amt transporter, is responsible for uptake of either ammonium or ammonia. The operon glnK-amtB is expressed in nitrogen-limiting conditions and is dependent on the NtrC regulatory protein. Two other genes talA and tktB are instead involved in the pentose phosphate pathway (non-oxidative branch).

Cluster n. 4 (of 27 genes) The genes in this cluster tend to have some basic pairwise association. The two genes rhsB and rhsA are homologous; yhfU and yhfV (php) are neighbors; frvB, frvX, are part of the frvABRX TU, glvG and glvC belong to glvBCG-ysdC. Three units of the TU yiaKLMNO-lyx-sgbHUE are co-clustered with the nearby genes yiaT and yiaW. The majority of the genes is involved in carbohydrate catabolism.

Cluster n. 5 (of 24 genes) Of the 24 genes of this cluster, 6 lie on the same operon trpLEDCBA and constitute the basic set of enzymes for the tryptophan biosynthesis pathway. Other 8 genes of this cluster belong to the transcription unit hisLGDCBHAFI (missing is hisL, closest to the promoter) and code for the 9 enzymes of the histidine biosynthesis pathway.

Cluster n. 6 (of 21 genes) Seven of the 8 subunits of the ATPase transcription unit atpBEFHAGDC are co-clustered. The missing gene, atpC, is the one closest to the downstream transcriptional terminator.

Cluster n. 7 (of 21 genes) It contains the leuLABCD operon, which encodes the enzymes responsible for the leucine biosynthesis pathway. It also contains the genes coding for the enzymatic complex acetolactate synthase/acetohydroxybutanoate synthase involved in the isoleucine biosynthesis from threonine. The following genes of this cluster are activated by the TF lrp (also in the cluster): serA, serC, ilvH, ilvI, aroA. This is one of the few clusters in which a TF and its target genes show similar expression patterns.

Cluster n. 8 (of 20 genes) The acetoacetyl-CoA transferase complex involved in acetoacetate degradation, is fully included; hyfH, hyfE, hyfD are part of the hydrogenase 4 complex, while yrfC and yrfD are involved in utilization of DNA as a carbon source.

Cluster n. 9 (of 20 genes) Most of the genes from the cystein regulon are present in the cluster (units cysDNC, cysIJH, cysK and most of cysPUWAM, all having the same regulator). They act as enzymes along the entire sulfate reduction I (assimilatory) pathway and form many complexes related to sulfur metabolism (sulfate adenylyltransferase, sulfite reductase-(NADPH), sulfate and thiosulfate ABC transporters). Notice that this cluster contains fliY.

Cluster n. 10 (of 18 genes) The rpl and rps genes are distributed in mainly 3 clusters (10, 20, and 25). In this cluster we find the first 7 genes of the operon rplNXE-rpsNH-rplFR-rpsE-rpmD-rplO-secY-rpmJ and the operon rpsJ-rplCDWB-rpsS-rplV-rpsC-rplP-rpmC-rpsQ (except for the last 2 genes). The rpl genes form the 50S ribosomal unit while rps the 30S unit. The two genes tufA and tufB which complete the cluster encode for the elongation factor Tu also involved in translation.

Cluster n. 11 (of 17 genes) The expression of the TU yciGFE, fully included in the cluster, is induced by osmotic stress. Correlated genes are involved in oxidoreductase, like wrbA, sodC (superoxide dismutase precursor), osmB (encoding an osmotically inducible lipoprotein), osmC (osmotically inducible peroxidase), yajO (putative NAD(P)H-dependent xylose reductase), otsA (a trehalose-6-phosphate synthase) and b1955, coding for a predicted phosphatase.

Cluster n. 12 (of 14 genes) Ten out of the 14 genes of this cluster belong to the same TU: rfaQGPSBI-JYZK.

Cluster n. 13 (of 14 genes) yhfG and fic belong to the yhfG-fic-pabA TU.

Cluster n. 14 (of 14 genes) The enterobactin synthase multienzyme complex is fully included in this cluster; fepA and fepB are part of the Ferric Enterobactin Transport System, and in fact the enterobactin biosynthesis pathway is fully represented by all its 6 genes in this cluster. FhuE is functionally related as it serves as a receptor for ferric-coprogen and ferric-rhodotorulic acid. Fiu (B0805) is a putative outer membrane porin like CirA. An interesting aspect is that the substrate spectrum of CirA is very similar to that of Fiu and this can explain the strong correlation. 2,3-Dihydroxybenzoate-AMP ligase, the entE gene product, activates DHB that participates to the enterobactin biosynthetic pathway, of which the final steps were thought to be carried out by a complex of the entE, F, D and B/G gene products. According to the cluster it is resonable that these genes are on the same pathway. Finally ybdB and entC, two other genes included in the cluster, belong to the same TU, entCP.

Cluster n. 15 (of 13 genes) phnJ, phnH, phnL, phnI, phnG, part of the phnCDEEFGHIJKLMNOP TU, are included in this cluster. Other genes are involved in oxidoreductase: yjjN (predicted L-galactonate oxidoreductase), yhcC (predicted Fe-S oxidoreductase), yjgI (predicted oxidoreductase with NAD(P)-binding Rossmann-fold domain).

Cluster n. 16 (of 13 genes) All the four genes involved in dTDP-L-rhamnose biosynthesis I (TU rfbDACX), belong to the cluster, along with the TU wbbIJH and wbbK. These TU share O-antigen functionalities.

Cluster n. 17 (of 13 genes) abgABT-ogt is the only complete TU included in the cluster. All the genes in this cluster are closely located on the genome although they do not seem to share any type of biological function.

Cluster n. 18 (of 13 genes) The 13 genes in this cluster belong to the operon nuoABCEFGHILMN and code for NADH dehydrogenase I.

Cluster n. 19 (of 12 genes) The sufABCDSE operon encodes components of a secondary pathway of iron-sulfur cluster assembly (ydiC is sufA, ynhA is sufE, b1680 is sufS). The other genes (b1747, b1596, b1745) in the cluster are involved in biosynthesis, metabolism and catabolism of arginine, playing an important role in cell division.

Cluster n. 20 (of 12 genes) All genes of this cluster are ribosomal rpl, rpm, rpo and rps.

Cluster n. 21 (of 12 genes) The liv family of genes from the (closely located) operons livKHMGF and livJ are co-clustered. They form the branched chain amino acids ATP Binding Cassette (ABC) transporter and leucine ABC transporter complexes, two ATP-dependent high-affinity branched-chain amino acid transport systems, and are members of the ABC superfamily of transporters. The operon thiCEFSGH is also contained in this cluster (except for thiS) and part of it forms the thiamin biosynthesis complex (thiG and thiH).

Cluster n. 22 (of 11 genes) There are two predicted oxidoreductases, Zn-dependent and NAD(P)-binding: yahK and yncB. The others genes seem to have no common functions.

Cluster n. 23 (of 11 genes) rfe, wzzE, wecB and rffG belong to the same TU: rfe-rffACDEGHMT-wzxE-wzyE-wzzE. Moreover there are two contiguous genes located near the TU, gppA and rhlB. To this cluster belong also: yhhF, encoding a 16S rRNA m2G966 methyltransferase, and sun, encoding a 16S rRNA m5C967 methyltransferase.

Cluster n. 24 (of 10 genes) This cluster is very neatly associated with the SOS pathway. The operon lexA-dinF, contained in this cluster, is involved in the SOS response, and so are dinD, dinI, oraA (i.e., recX), recA, recN, sulA, yebG. Notice that dinF, recA, sulA, oraA and recN are all under transcriptional regulation of lexA.

Cluster n. 25 (of 10 genes) This is another functionally compact cluster (ribosomal genes). We find here the rpl part of rplKAJL-rpoBC, together with other rps genes (see cluster 10). Of the two non-ribosomal genes in the cluster one, priB, is contained in the ribosomal protein operon rpsF-priB-rpsR-rplI.

Cluster n. 26 (of 10 genes) The galETKM and the araBAD operons form this cluster. This last form the L-arabinose degradation pathway. Notice that the low-affinity proton-driven transporter, araE, one of the mechanisms by which L-arabinose enters *E.coli*, also is in the cluster.

Cluster n. 27 (of 10 genes) In the same cluster we have: 50S ribosomal subunit protein L27 (rpmA), 50S ribosomal subunit protein L21 (rplU), 30S ribosomal subunit protein S20 (rpsT).

Cluster n. 28 (of 10 genes) Four of the 10 genes code for predicted prophage proteins.

Cluster n. 29 (of 10 genes) Six of the genes are transposon-related: yi22-2, yi22-1, yi82-1, yi22-4, tra5-3 and tra5-1. In addition there are two contiguos genes : yfhA and yfhG.

Cluster n. 30 (of 10 genes) yahF, yahI belong to yahDEFG, while hcaA2 and hcaC belong to hcaEFCBD. Finally b2359 codes for a CPS-53 (KpLE1) prophage predicted protein, ybcO for a DLP12 prophage predicted protein, and ykfF for a CP4-6 prophage predicted protein.

Cluster n. 31 (of 10 genes) yfaP and yfaS-1 belong to yfaAPQS-1S-2T TU, in addition to wcaL, encoding for a predicted colanic biosynthesis glycosyl transferase, yfcC, a putative S-transferase and yeaV, a predicted transporter.

Cluster n. 32 (of 10 genes) pyrF-yciH belongs to this cluster together with pyrD.

Cluster n. 33 (of 10 genes) This cluster contains the hyc operon hycABCDEFGHI and the two related complexes formate hydrogenlyase and hydrogenase 3.

Cluster n. 34 (of 10 genes) The TU sdhCDAB-sucABCD,which is part of the aerobic respiratory chain (through succinate dehydrogenase) and part of the Krebs cycle (through succinyl-CoA synthetase), is entirely present in this cluster with 2 other genes: b0725 and ndk. The first one is considered a phantom gene, while the second one catalyzes the reaction in which the terminal phosphate of a nucleoside-triphosphate is transferred to a nucleoside-diphosphate. The clustering suggests that nucleoside-diphosphate kinase be involved in the synthesis of ATP, an hypothesis considered unlikely in the literature.

Cluster n. 35 (of 10 genes) yibHI is fully included together with the neighbouring gene mtlA.

Cluster n. 36 (of 9 genes) Eight of the 9 genes in this cluster are arg genes forming the backbone of the arginine biosynthesis pathway. The remaining gene artJ (or argR) also binds arginine.

Cluster n. 37 (of 9 genes) Four genes belong to the appCBA-yccB TU, that is co-clustered with the adjacent TU hyaABCDEF (all genes are present).

Cluster n. 38 (of 8 genes) Most of the genes in this clusters (gadA, gadB, hdeA, hdeB, hdeD) are transcriptionally regulated by yhiE (i.e., gadE), which also belongs to the cluster.

Cluster n. 39 (of 8 genes) It contains the operon srlAEBD-gutM-srlR-gutQ (except the last two genes). GutM is also a transcription factor for this operon.

Cluster n. 40 (of 8 genes) hemG and yigZ take part to the same transcription unit of trkH: hemG-pepQ-trkH-yigZ. Of the 2 TU that can be associated with hemC, hemCDXY and hemC-nfi-nudC, we find here evidence for the second one (the gene nfi is in the cluster). The remaining genes rfaF and rfaD are part of the rfaDFCL TU.

Cluster n. 41 (of 8 genes) All genes code for tRNA: 2 leucine tRNAs, 2 valine tRNAs, the 2 phenylalanine tRNAs, one of seven arginine tRNAs, and the sole cysteine tRNA.

Cluster n. 42 (of 8 genes) NA

Cluster n. 43 (of 8 genes) Two probes are referring to the same gene yeaL coding for a conserved inner membrane protein, while YehY is a membrane component of an ABC transporter involved in osmoprotection.

Cluster n. 44 (of 8 genes) The TU malPQ, malK-lamB-malM and malEFG belong to the cluster. They code for the Maltose-Maltodextrine transport system.

Cluster n. 45 (of 7 genes) uidB and uidC belong to the same TU uidABC.

Cluster n. 46 (of 7 genes) sgaB and sgaE, whose products are involved in utilization of L-ascorbate, belong to ulaABCDEF.

Cluster n. 47 (of 7 genes) The intE-ymfGH transcription unit is included in this cluster. The function of these 7 genes is unknown.

Cluster n. 48 (of 7 genes) The TU fdoGHI-fdhE and treBC are fully included in the cluster.

Cluster n. 49 (of 7 genes) The genes rhaD and rhaB belong to rhaBAD, and frvA is positioned close to this transcription unit. yjcV and yjcW belong to alsRBACE and, as frvA, are involved in carbohydrate catabolism.

Cluster n. 50 (of 7 genes) NA

Cluster n. 51 (of 7 genes) yeiM codes for a NUP transporter, ttdT for a tartrate/succinate DASS transporter (YgjE) and mhpT for a MFS transport.

Cluster n. 52 (of 7 genes) NA

Cluster n. 53 (of 7 genes) Most of the products of the genes belonging to the cluster are involved in the biosynthesis of proteins, like a protein translation (Pth), and a nucleobase and nucleotide interconversion (Tdk).

Cluster n. 54 (of 7 genes) cbpAM belongs to this cluster, in addition to ybaST and the gene just following this TU on the chromosome.

Cluster n. 55 (of 7 genes) sbm and ygfD belong to scpA-argK-scpBC, with two contiguos genes belonging to two different TU.

Cluster n. 56 (of 7 genes) The carAB operon is co-clustered with aspartate carbamoyltransferase and with pyrC. Notice that the 3 compounds are neighbouring enzymes on the first part of the de novo biosynthesis of pyrimidine ribonucleotides. The remaining 2 genes codAB are also involved in pyrimidine ribonucleotides pathways and in pyrimidine transporters in general.

Cluster n. 57 (of 7 genes) The TU hypABCDE is fully included in the cluster (except hybE) and is involved in the maturation of hydrogenase isozymes. nirB and nirD are part of nirBDC-cysG TU involved in nitrite reductase. *E. coli* has two distinct nitrite reductases. Their expression is complementary: with low concentrations of nitrate in the environment NrfA (cluster 246) is expressed; with high concentrations of nitrate NirB is expressed almost exclusively. The clustering maintains this difference.

Cluster n. 58 (of 7 genes) The arnABCT-yfbHJW TU, except for arnT (b2257), is belonging to the cluster with a neighbouring gene, b2252, induced by aluminum, that can interact also with the remaining gene, a predicted cytochrome (b1974).

Cluster n. 59 (of 7 genes) metT, metU and leuW belong to the same Transcription unit: metT-leuW-glnUW-metU-glnVX. argZ, argQ and argV belong to serV-argVYZQ, while argX to argX-hisR-leuT-proM.

Cluster n. 60 (of 7 genes) aspV, aspU, and aspT belong to the cluster.

Cluster n. 61 (of 7 genes) All the genes have the same prefix rrf, and are involved in the biosynthesis of ribosomal RNA.

Cluster n. 62 (of 7 genes) gatYZABCD is fully included in this cluster.

Cluster n. 63 (of 6 genes) yeeT and yeeV are part of the yeeRSTUVW TU (CP4-44 prophage). YfdO is a predicted defective phage replication protein O (CPS-53 (KpLE1) prophage).

Cluster n. 64 (of 6 genes) ycbT, ycbF and ycbV belong to ycbFRSTUV, etp and etk belong to etk-etp-gfcE.

Cluster n. 65 (of 6 genes) NA

Cluster n. 66 (of 6 genes) It contains glpG which is part of the glpEGR operon. The other two genes are not included. The discovery of multiple promoters responsible for the expression of glpE, glpG and glpR can provide a possible reason for the weak correlation. The truB, nusA, infB, yhbC genes are all included in the metY-yhbC-nusA-infB-rbfA-truB-rpsO-pnp transcription unit. In particular truB contributes to thermal stability of tRNA and nusA in both prevention and enhancement of transcriptional termination. IF-2 (infB) is one of three translation initiation factors in *E.coli*. MioC is suggested to play a role in electron transport during catalysis of the biotin synthase reaction, and biotin itself is known to biotinylate histones and posttranslational modifications of histones, processes involved in regulation of gene expression and DNA repair.

Cluster n. 67 (of 6 genes) dmsB and dmsA are two components of the dmsABC transcription unit (dmsC is missing). Dimethyl sulfoxide (DMSO) reductase is a terminal electron transfer enzyme, catalyzing a step in an energy-transducing anaerobic electron transport chain. The enzyme functions anaerobically in the absence of nitrate. ynfF (b1588) and ynfE (b1587) are highly similar to dmsA (paralogs), while ynfG is highly similar to dmsB. Little is known about ydfZ (b1541). From previous analysis it seems involved in electron transfer during anaerobic growth, and the cluster reinforces this assumption.

Cluster n. 68 (of 6 genes) These genes seem to have non particular common features. Worth mention is fhiA, which represents the fused boundaries of a 44 gene cluster encoding a second flagellar system designated Flag-2 in *E.coli* strain 042. The Flag-2 system has been deleted in K-12 leaving the fhiA-mbhA gene cluster promotorless.

Cluster n. 69 (of 6 genes) The metNIQ TU is fully included in the cluster with metA, metF and metE.

Cluster n. 70 (of 6 genes) NlpC is a putative lipoprotein hydrolase, YcgM a predicted isomerase/hydrolase, YdhO a putative lipoprotein. Cls is involved in cardiolipin synthase, one of the major membrane phospholipids, while the ribE gene encodes lumazine synthase, an enzyme that catalyzes the penultimate step in the riboflavin biosynthesis pathway.

Cluster n. 71 (of 6 genes) yrfG and yrfH compose the hslR-yrfG TU, while hslO (yrfI), immediately following it on the genome, forms the molecular chaperone Hsp33, and hslV is part of the hslVU protease. The gene tusB belongs to tusBCD-yheO and is involved in the sulfur transfer protein complex. dsbC is involved in chaperon, repair (refolding) as well as yrfH and yrfI.

Cluster n. 72 (of 6 genes) The genes form the ilvLGMEDA operon (except for ilvL and ilvG_2), and enter (with 6 out of 10 enzymes) into the isoleucine biosynthesis from threonine pathway.

Cluster n. 73 (of 5 genes) NA

Cluster n. 74 (of 5 genes) RhaT is the sole transporter for rhamnose in *E.coli* and functions as a rhamnose/proton symporter. The gene rhaT is included in the cluster with his regulator RhaS.

Cluster n. 75 (of 5 genes) The genes rpsB and tsf form the rpsB-tsf TU coding for the 30S ribosomal subunit protein S2. b3836 is part of the tatABC TU and b3837 is a putative histon, while pyrH plays a role in the transcriptional regulation of the carAB operon included in cluster 57, highlighting once again the fact that transcriptional regulation cannot be recovered with so much precision.

Cluster n. 76 (of 5 genes) YciW is a predicted oxidoreductase protein, while cysM is part of the cysPUWAM TU. Two remaing genes, gsiA and gsiB, form the gsiABCD-iaaA TU, involved in glutathione ABC transporter, similarly to B1729 which is classified as a predicted transporter.

Cluster n. 77 (of 5 genes) NA

Cluster n. 78 (of 5 genes) The genes yddO, yddP, yddQ, yddR, and yddS are probably located within a single operon. Based on sequence similarity, these proteins probably function together as an ATP-dependent peptide transporter.

Cluster n. 79 (of 5 genes) The genes b1440, b1442 and b1443 form the ydcSTUV TU, and they may function together as an ATP-dependent spermidine/putrescine transporter.

Cluster n. 80 (of 5 genes) The level of OsmY is sensitive to hyperosmotic stress, similarly to OsmE that is an osmotically inducible gene product. Correlated to this protein not only in terms of their expression pattern but also of function is Dps that was shown to be involved in protection from multiple stresses, including oxidative stress. Finally ybaY and ybjP are predicted to code for a lipoprotein.

Cluster n. 81 (of 5 genes) YjbJ is predicted to be a stress response protein, while the other 4 genes form the yqjCDEK TU.

Cluster n. 82 (of 5 genes) ntpA, involved in nucleobase, nucleoside and nucleotide interconversion, is part of the nudBp TU. Related to this gene is nudK, coding for a nucleotide-sugar hydrolase. ArgS is a member of the family of aminoacyl tRNA synthetases, as AspS.

Cluster n. 83 (of 5 genes) HisS is a member of the family of aminoacyl-tRNA synthetases. rpsM and D take part to the rpsMKD-rpoA-rplQ. SecY (prlA) and SecE are inner membrane proteins involved in the Sec protein secretion pathway.

Cluster n. 84 (of 5 genes) The nlpD-rpoS TU is included in this cluster.

Cluster n. 85 (of 5 genes) PotFGHI is an ATP-dependent putrescine transporter similar in sequence and subunit composition to the PotABCD putrescine/spermidine uptake system (cluster 157). The fact that these two complexes are in distinct clusters suggests an activation due to different environmental conditions.

Cluster n. 86 (of 5 genes) This cluster seems to be very heterogenous from a point of view of functions, in fact yecK and bisZ form the torYZ TU that represents a third TMAO respiratory system in *E.coli*, while ydeE may function as a proton-driven drug efflux system (the ydeE gene probably constitutes a monocistronic operon) and alkA is involved in DNA repair.

Cluster n. 87 (of 5 genes) YbfD and YbfL are respectively putative DNA ligase and transposase, as YhhI. The b4285 is a partial transposase. No informations are provided for the ygdB gene, but the compactness of the cluster suggest a role in transposase.

Cluster n. 88 (of 5 genes) purE and purK take part to the purEK TU. purF with cvpA belong to cvpA-purF-ubiX while purM belongs to a different TU (purMN). All the genes are involved in purine nucleotide biosynthesis.

Cluster n. 89 (of 5 genes) The TU yrbBCDEF has 3 genes in this cluster (D-E-F subunits). The yrbF and yrbE genes may function together as an ATP-dependant toluene efflux transporter. It is also possible that either YrbD or YrbC might be the periplasmic binding protein of this complex. The clustering suggests that YrbD is more likely to fulfill this role (yrbC, in fact, co-clusters with yrbB in cluster 360).

Cluster n. 90 (of 5 genes) The aroD and aroH gene products are involved in the chorismate biosynthesis.

Cluster n. 91 (of 5 genes) The TU yrdD-rimN-aroE-yrdB accounts for 3 genes in this cluster yrdD, aroE and yrdB all involved in shikimate dehydrogenase. yrdB follows aroE on the genome and it has unknown function.

Cluster n. 92 (of 5 genes) basR and basS form the basRS TU involved in transcriptional regulation.

Cluster n. 93 (of 5 genes) This cluster contains genes b2970 to b2974. Each gene is annotated as forming a single gene operon, and for most of them there is no information about the biological function. This result suggests that they may be part of the same operon.

Cluster n. 94 (of 5 genes) RecJ is involved in DNA catabolism and is essential for the initiation of recombination. dnaA and dnaN are part of the dnaAN-recF TU. The remaining two genes amiB and mutL are part of the same TU yjeFE-amiB-mutL-miaA-hfq-hflXKC involved in cell division.

Cluster n. 95 (of 5 genes) speG and ynfB form the speG-ynfB TU.

Cluster n. 96 (of 5 genes) Four out of the five genes present in this cluster are part of the paaABCDE-FGHIJK TU involved in phenylacetate catabolism.

Cluster n. 97 (of 5 genes) The genes that form the kdpFABC TU are all but one included in this cluster. The kdpFABC genes encode an ATP-dependent potassium ion transporter. KdpC is involved in linking KdpA and KdpB together while the fourth gene on the same operon, kdpF (the one missing), encodes a small non-essential polypeptide, which was shown to be associated with and stabilize the KdpFABC complex in vivo. In this case the relationship between the genes that form the complex is stronger than that between the genes of the TU.

Cluster n. 98 (of 5 genes) The cluster includes all the 4 genes of narGHJI plus narK. NarK is a nitrite extrusion protein involved in anaerobic nitrate respiration. Nitrate is the preferred electron acceptor for anaerobic respiration in *E.coli*, resulting in reduction to nitrite, which is either excreted or further reduced. *E.coli* contains three nitrate reductases. Two of them, nitrate reductase A (NRA) and nitrate reductase Z (NRZ), are membrane bound and biochemically similar. The third nitrate reductase, Nap (cluster 70), is located in the periplasm. Nitrate reductase A is expressed when levels of nitrate in the environment are high, Nap is expressed when they are low, while NRZ expression is not dependent on nitrate levels or anaerobiosis, but induced during stationary phase.

Cluster n. 99 (of 5 genes) All the 5 genes of dppABCDF are present in the cluster. DppABCDF is similar in sequence and subunit composition to the oligopeptide uptake system OppABCDF (oppA-B included in cluster 414, oppC-F-D in cluster 240), suggesting similar subunit functions, although they may function under different conditions.

Cluster n. 100 (of 5 genes) lysT-valT-lysW is represented in this cluster by lysT and lysW; lysY is part of the lysY-valZ TU while lysZ and lysQ are in the same operon. The genes code for 5 of the 6 lysine tRNAs.

Cluster n. 101 (of 5 genes) The CusCFBA copper efflux system is completely included in the cluster along with its transcriptional regulator YlcA.

Cluster n. 102 (of 5 genes) This cluster contains all the four asparagine tRNAs. The remaining gene is serW, one of five serine tRNAs.

Cluster n. 103 (of 5 genes) b2603 and b2604 belong to the same TU for which no information has been found.

Cluster n. 104 (of 5 genes) The TU cyoABCDE is included in the cluster.

Cluster n. 105 (of 5 genes) All the 5 genes of the NikABCDE ATP-dependent nickel (II) uptake system belong to the cluster.

Cluster n. 106 (of 5 genes) The products of cspA, cspG and cspH are all part of the CspA family of proteins, related to response to temperature stimuli, just like another gene belonging to this cluster (ddg).

Cluster n. 107 (of 5 genes) The five genes are all from the operon moaABCDE. MoaD and MoaE form the molybdopterin synthase enzymatic complex.

Cluster n. 108 (of 5 genes) All the 5 genes are related to the glycerol system. glpT and glpQ belong to the glpTQ TU that is the major *E.coli* uptake system for glycerol-3-phosphate. The remaining genes form the glpFKX transcription unit involved in facilitating diffusion of glycerol into the cell.

Cluster n. 109 (of 5 genes) This cluster contains the rbsDACBKR TU, an ATP-dependent ribose transporter.

Cluster n. 110 (of 5 genes) The transcription unit secE-nusG, and the second half of the transcription unit thrU-tyrU-glyT-thrT appear in the cluster (the other two genes have a lower correlation).

Cluster n. 111 (of 5 genes) The genes form the garPLRK-rnpB TU (rnpB is missing).

Cluster n. 112 (of 5 genes) It contains the entire operon fecABCDE.

Cluster n. 113 (of 4 genes) It contains genes yhiU and yhiV, components of the MdtEF multidrug transporter. For the other two genes less is known.

Cluster n. 114 (of 4 genes) NA

Cluster n. 115 (of 4 genes) The TU yghDE (coding for two predicted secretion pathway proteins) is included in this cluster with YhdJ, a predicted methyltransferase.

Cluster n. 116 (of 4 genes) All the four genes belong to the same TU: ycfK-ymfLMNOPQRST.

Cluster n. 117 (of 4 genes) fixC and fixB belong to fixABCX TU, probably involved in anaerobic metabolism of carnitine. This hypothesis is reinforced by the cluster which contain also caiD, part of caiTABCDE, that has carnitine racemase activity.

Cluster n. 118 (of 4 genes) The genes belong to four different TU, although yibJ and yibG are closely located on the genome.

Cluster n. 119 (of 4 genes) spoU, spoT and recG all belong to the same TU, rpoZ-spoT-trmH-recG.

Cluster n. 120 (of 4 genes) NA

Cluster n. 121 (of 4 genes) YjhD, YjhE and Yi91b are KpLE2 phage-like elements.

Cluster n. 122 (of 4 genes) fcl, gmd and wcaI belong to the same TU, cpsBG-fcl-gmd-nudD-wcaDEFIJ-wzxC.

Cluster n. 123 (of 4 genes) tusC-D and yheO are part of the tusBCD-yheO TU.

Cluster n. 124 (of 4 genes) NA

Cluster n. 125 (of 4 genes) NA

Cluster n. 126 (of 4 genes) ubiE and ubiD are included in this cluster.

Cluster n. 127 (of 4 genes) UbiG is an O-methyltransferase that catalyzes both O-methylation reactions in the biosynthesis of ubiquinone, while YdgJ , based on sequence similarity, is predicted to be a D-galactose 1-dehydrogenase. The remaining genes are closely positioned on the genome, one is a predicted acyl-CoA dehydrogenase (b1695), the other a putative subunit of YdiQ-YdiR flavoprotein.

Cluster n. 128 (of 4 genes) gltD and gltB are part of gltBDF, operon which comprises genes coding for the large (gltB) and small (gltD) subunits of the glutamate synthase enzyme. Asd is an aldose sugar dehydrogenase, while LysC is involved in the phosphorylation of aspartate, the first step in the biosynthesis of 4 different amino acids: lysine, methionine, threonine, and isoleucine.

Cluster n. 129 (of 4 genes) SgaU is a L-xylulose 5-phosphate 3-epimerase, YhgJ a RNA 3'-terminal phosphate cyclase, and the 2 remaining genes are transcriptional regulators.

Cluster n. 130 (of 4 genes) NA

Cluster n. 131 (of 4 genes) NA

Cluster n. 132 (of 4 genes) YhfP is a fructoselysine 3-epimerase; near to it on the genome is yhfS. YphE is a predicted ATP-dependent sugar transporter.

Cluster n. 133 (of 4 genes) b2350 and b2351 are part of yfdGHI, while speD and speE, both involved in polyamine biosynthesis, are part of yacC-speED TU.

Cluster n. 134 (of 4 genes) ygeG, pbl and ygeL are closely located on the genome.

Cluster n. 135 (of 4 genes) rplS and rplI are two 50S ribosomal subunit proteins.

Cluster n. 136 (of 4 genes) MltD is a membrane-bound lytic murein transglycosylase. The tgt gene encodes tRNA-guanine transglycosylase.

Cluster n. 137 (of 4 genes) The operon deoCABD, included, codes for part of the degradation of pyrimidine deoxyribonucleosides pathway.

Cluster n. 138 (of 4 genes) NA

Cluster n. 139 (of 4 genes) proWVX is a transcription unit (represented by 3 out of the four genes), involved in the transport system for the osmoprotectant glycine betaine. The intracellular accumulation of glycine betaine by *E.coli* permits growth in a high-osmolarity environment. The remaining gene ygaY (with ygaX), appear to be one of the two segments of a gene encoding an uncharacterized permease of the major facilitator superfamily (MFS) of transporters. The interesting aspect is that ygaY was shown to be induced by osmotic upshift. This provides evidence of a tight relationship with the proWVX system.

Cluster n. 140 (of 4 genes) The cluster contains the periplasmic nitrate reductase Nap. NapD has an undefined role in the post-translational assembly of a functional NapA, and it is essential for Nap activity. NapF, although not essential, stimulates its activity; finally NapF does not appear to be involved in the electron transfer. It has been argued that NapF may play a role in energy conservation rather than a direct role in nitrate reduction.

Cluster n. 141 (of 4 genes) NA

Cluster n. 142 (of 4 genes) RecQ is an ATP-dependent DNA helicase that functions by blocking illegitimate recombinations, enhancing topoisomerase activity, initiating SOS signaling and clearing blocked replication forks. Interestingly in the same cluster there is DnaB, the replicative DNA helicase, that progressively unwinds DNA at replication forks in advance of DNA polymerase, suggesting a cooperation between the two genes.

Cluster n. 143 (of 4 genes) yjfM and yjfL belong to yjfCKLM and YjjQ is a predicted DNA-binding transcriptional regulator.

Cluster n. 144 (of 4 genes) DgoK takes part in a pathway for the catabolism of galactonate. AgaS has been predicted to be an isomerase involved in N-acetyl-galactosamine and galactosamine utilization while the alsK gene encodes a putative D-allose kinase.

Cluster n. 145 (of 4 genes) Three out of four genes in the cluster belong to gspCDEFGHIJKLMO TU.

Cluster n. 146 (of 4 genes) NA

Cluster n. 147 (of 4 genes) The gspA-pioO TU is included in the cluster. yicO and uhpC are located near on the genome.

Cluster n. 148 (of 4 genes) GatR is a transcriptional repressor, while YcfT and FocB are protein transporters. The remaining element is an unknown function gene.

Cluster n. 149 (of 4 genes) yrbG and yrbH are two consecutive genes on the genome. The other two genes seem to have no common feature.

Cluster n. 150 (of 4 genes) pepP and ygfB are part of the same TU: pepP-ubiH-visC-ygfB.

Cluster n. 151 (of 4 genes) NA

Cluster n. 152 (of 4 genes) NA

Cluster n. 153 (of 4 genes) The bcsEFG TU is fully included.

Cluster n. 154 (of 4 genes) The fgfTS TU is present in the cluster, with an a-glycosidase and a probable pyruvate formate lyase 2 activating enzyme (PflC).

Cluster n. 155 (of 4 genes) These genes form the bioBFCD transcription unit for biotin synthase.

Cluster n. 156 (of 4 genes) The four genes seem to have no common features, although bglF and yieC are closely located on the genome.

Cluster n. 157 (of 4 genes) potA-B-C-D are all required for polyamine transport and are included in potABCD transcription unit.

Cluster n. 158 (of 4 genes) All the genes belong to the same TU: ydjXYZ-ynjABCD.

Cluster n. 159 (of 4 genes) It contains the csgDEFG operon, involved in the Curli Secretion and Assembly Complex.

Cluster n. 160 (of 4 genes) NA

Cluster n. 161 (of 4 genes) sgcQ, sgcX and sgcA are part of sgcABC unit, a putative PTS permease, belonging to the functional superfamily of the phosphoenolpyruvate (PEP)-dependent, sugar transporting phosphotransferase system (PTS). The function of these proteins is not known, but they may function in the transport and phosphorylation of 5-carbon sugars. YtfR, YtfS, YjfF, YtfT (the only included in this cluster), and YtfQ are uncharacterized members of the ABC superfamily of transporters. Based on sequence similarity they probably function together as an ATP-dependant sugar transporter. This cluster reinforce this assumption. (see also cluster 163)

Cluster n. 162 (of 4 genes) NA

Cluster n. 163 (of 4 genes) YtfR is an ATP-binding component of a predicted sugar ABC transporter. SgcA is included in the phosphoenolpyruvate-dependent sugar phosphotransferase system and YghK, also named GlcA, is a glycolate transporter. UgpA is part of the UgpABCE glycerol-3-phosphate uptake system, and is also an essential intermediate in phospholipid biosynthesis. The roles of the entire set of genes are closely related and suggest the involvement in the same pathway to using Glycerol-3-phosphate as a carbon source and a phosphate source.

Cluster n. 164 (of 4 genes) The araFGH operon codes for the arabinose ABC transporter.

Cluster n. 165 (of 4 genes) The nrdHIEF TU is fully included in this cluster.

Cluster n. 166 (of 4 genes) The operon metZWV co-clusters with metY. Of the 6 methionine tRNAs, these 4 have in common that they operate during initiation of protein synthesis rather than protein elongation.

Cluster n. 167 (of 4 genes) The operon glyVXY and glyW code for 4 of the 6 glycine tRNAs.

Cluster n. 168 (of 4 genes) The leuQPV TU is belonging to this cluster in addiction to leuT, belonging to a different TU. The genes encode 4 of the 8 leucine tRNAs.

Cluster n. 169 (of 4 genes) The four genes are consecutive on the genome but are part of three different TU.

Cluster n. 170 (of 4 genes) The 4 genes are all related to glycogen metabolism. GlgA and glgC belong to the glgCAP TU, glgX to glyVXY, and glgB to glgB.

Cluster n. 171 (of 4 genes) This cluster contains 4 of the 7 genes coding for valine tRNAs.

Cluster n. 172 (of 4 genes) It contains the (Fur-regulated) exbBD operon and the exbBD part of various complexes. This is co-clustered with two other Fur-regulated genes (fhuF and fecI).

Cluster n. 173 (of 4 genes) ileU and alaU belong to the rrsD-ileU-alaU-rrlD-rrfD-thrV-rrfF TU, while ileT and alaT to rrsA-ileT-alaT-rrlA-rrfA. All participate to triplet codon-amino acid adaptor activity.

Cluster n. 174 (of 4 genes) All the genes belong to tdcABCDEFG TU.

Cluster n. 175 (of 4 genes) This cluster contains the 4 genes for the glutamate tRNAs.

Cluster n. 176 (of 4 genes) The 4 genes of the operon iscRSUA form the cluster. The gene iscR (b2531) is also a transcriptional regulator for the operon.

Cluster n. 177 (of 4 genes) NA

Cluster n. 178 (of 4 genes) rsxA-B-D take part to the rsxABCDGE operon and co-cluster with the upstream gene cnu (b1625).

Cluster n. 179 (of 4 genes) hscBA-fdx is fully included in this cluster in association with the gene preceding it on the genome.

Cluster n. 180 (of 3 genes) ydcP is a putative collagenase and it is a single gene TU, the other two genes form the ansA-pncA TU.

Cluster n. 181 (of 3 genes) b0967 is predicted methyltransferase. Its gene is located close to yccF.

Cluster n. 182 (of 2 genes) NA

Cluster n. 183 (of 3 genes) yjeQ, a ribosome small subunit-dependent GTPase, and Psd, a phosphatidylserine decarboxylase, proenzyme, belongs to two different TU, although they are neighbors on the genome.

Cluster n. 184 (of 3 genes) proVWX, the high-affinity transport system for the osmoprotectant glycine betaine, is represented here by proW and proX.

Cluster n. 185 (of 3 genes) Two out of the 3 genes included in this cluster form the kbl-tdh TU.

Cluster n. 186 (of 3 genes) yafO and P are part of the yafNOP TU.

Cluster n. 187 (of 3 genes) NA

Cluster n. 188 (of 3 genes) All the probes are referring to IS1, the smallest insertion sequence in *E.coli*, that codes for three proteins, InsA, InsB and InsAB'.

Cluster n. 189 (of 3 genes) As the previous one, this cluster has 2 probes that refer to genes whose products are IS150 proteins InsB and InsA.

Cluster n. 190 (of 3 genes) NA

Cluster n. 191 (of 3 genes) aaB and maoC are part of, respectively, paaABCDEFGHIJK and maoC TU, but they are closely located on the genome.

Cluster n. 192 (of 3 genes) yeiP and pdxB code for two predicted dehydrogenases, while for ycaO no information has been found by a literature search.

Cluster n. 193 (of 3 genes) NA

Cluster n. 194 (of 3 genes) NA

Cluster n. 195 (of 3 genes) NA

Cluster n. 196 (of 3 genes) All the probes in the cluster are referring to the insC gene.

Cluster n. 197 (of 3 genes) NA

Cluster n. 198 (of 3 genes) Two genes, mreB and mreD, are responsible for rod shape and mecillinam sensitivity in *E.coli.* In particular MreB is responsible for proper chromosome segregation and movement and its overexpression can inhibit cell division. Yeast two-hybrid experiments have shown that MreBCD form a complex in which MreB interacts with MreC (not present), and MreC interacts with MreD. Such indirect relationship is represented in this cluster.

Cluster n. 199 (of 3 genes) NA

Cluster n. 200 (of 3 genes) NA

Cluster n. 201 (of 3 genes) yjdJ and yjdI are adjacent on the genome.

Cluster n. 202 (of 3 genes) cdd and udp have a common regulator, CytR, regulator for deo operon, udp, cdd, tsx, nupC, and nupG. The remaining gene is a transcriptional repressor for the glucitol utilization. It belongs to the DeoR family of transcriptional regulators.

Cluster n. 203 (of 3 genes) The three genes belong to different TU but are all involved in anaerobic respiration.

Cluster n. 204 (of 3 genes) NA

Cluster n. 205 (of 3 genes) Two genes form the yifNO TU, while the remaining is a putative replicase.

Cluster n. 206 (of 3 genes) NA

Cluster n. 207 (of 3 genes) NA

Cluster n. 208 (of 3 genes) NA

Cluster n. 209 (of 3 genes) hflD shares an operon with purB, which is involved in methionine synthesis.

Cluster n. 210 (of 3 genes) G6740 is a phantom gene, while the remaining genes are part of the wza-wzb-wzc-wcaAB TU.

Cluster n. 211 (of 3 genes) NA

Cluster n. 212 (of 3 genes) NA

Cluster n. 213 (of 3 genes) yahG is part of yahDEFG TU, located near the yagQRST TU, which contains the other two genes included in the cluster, yagQ and yagR.

Cluster n. 214 (of 3 genes) Genes ftsW and mraY are part of mraZW-ftsLI-murEF-mraY-murD-ftsW-murGC-ddlB-ftsQAZ transcriptional unit. FtsW is an essential cell division protein. yacC is cotranscribed with speED (feature not seen by our cluster) and is related to spermidine synthase and S-adenosylmethionine decarboxylase activity. This result may be in agreement with the discovery of [6], regarding the effect of spermidine on cell proliferation.

Cluster n. 215 (of 3 genes) NA

Cluster n. 216 (of 3 genes) yafWXY-ykfBFGHI includes two genes part of the cluster. The remaining gene is relatively close to the TU.

Cluster n. 217 (of 3 genes) YaiU seems to be a putative flagellin structural protein, although this hypothesis is not supported by the clustering.

Cluster n. 218 (of 3 genes) These genes form the rpsLG-fusA-tufA transcription unit.

Cluster n. 219 (of 3 genes) FrvR and RtcR are two transcriptional regulators, while HyuA has similarity to allantoinase enzymes.

Cluster n. 220 (of 3 genes) holD belongs to holD-rimI-yjjG TU. The remaining genes form sspAB TU.

Cluster n. 221 (of 3 genes) Three of the four genes forming prpBCDE are included in this cluster.

Cluster n. 222 (of 3 genes) The ymfDE TU is present in this cluster in addition to the lit gene located upstream of the TU.

Cluster n. 223 (of 3 genes) The pspA-B-C belong to the same TU: pspABCDE.

Cluster n. 224 (of 3 genes) NA

Cluster n. 225 (of 3 genes) NA

Cluster n. 226 (of 3 genes) NA

Cluster n. 227 (of 3 genes) The three genes belong to three different TU although surE and ygbB are closely positioned on the genome.

Cluster n. 228 (of 3 genes) The yfbTU TU is included in this cluster.

Cluster n. 229 (of 3 genes) The 3 genes form the glycine cleavage system, that is a multi-enzyme complex that catalyzes the reversible oxidation of glycine.

Cluster n. 230 (of 3 genes) ftsE and ftsY belong to ftsYEX.

Cluster n. 231 (of 3 genes) The aceEF TU belongs to this cluster. It forms part of the Pyruvate dehydrogenase multienzyme complex (which has a very complicated structure: 12 AceE dimers, a 24-subunit AceF core, and 6 LpdA dimers). This complex is co-clustered here with Ndh, a NADH dehydrogenase.

Cluster n. 232 (of 3 genes) Dihydroxyacetone kinase is composed of three subunits, all present in the cluster: dhaK, dhaL, and dhaM.

Cluster n. 233 (of 3 genes) narZ and narY are part of the narZYWV operon. (narW and narV are in cluster 243 and 71). While these two genes are involved in nitrate reductase, narU, the last gene in the cluster, is a nitrate extrusion protein.

Cluster n. 234 (of 3 genes) The genes form the manXYZ transcription unit. ManXYZ, the mannose PTS permease, belongs to the functional superfamily of the phosphoenolpyruvate-dependent, sugar transporting phosphotransferase system.

Cluster n. 235 (of 3 genes) NA

Cluster n. 236 (of 3 genes) yfbK is an unknown function gene, while the other two genes are included in the cpsBG-fcl-gmd-nudD-wcaDEFIJ-wzxC TU.

Cluster n. 237 (of 3 genes) NA

Cluster n. 238 (of 3 genes) The nrdAB TU and the gene located just upstream on the genome are included in this cluster.

Cluster n. 239 (of 3 genes) The hinT-nagZ-thiK-ycfLMP TU is represented here by the ycfL-M-P genes.

Cluster n. 240 (of 3 genes) oppC-D-F, included here, and oppA-B (in cluster 134) are part of the same operon OppABCDF. The system has been observed to function in oligopeptide uptake, as well as recycling of cell wall peptides.

Cluster n. 241 (of 3 genes) frmABR is fully included in the cluster. Transcription of the frmRAB operon is induced by formaldehyde.

Cluster n. 242 (of 3 genes) lsrACDBFG is represented by b1518 and b1516, in association with ydeV, a nearby gene.

Cluster n. 243 (of 3 genes) ymgAB is part of the cluster, with the gene just upstream ycgZ.

Cluster n. 244 (of 3 genes) The triplet frdA, frdB and frdD form the fumarate reductase complex. The forth element of the operon frdABCD (not involved in the protein complex) is missing from the cluster.

Cluster n. 245 (of 3 genes) Two genes belong to the puuDR TU. The third gene is the one located before the TU: puuA.

Cluster n. 246 (of 3 genes) These genes are part of the transcription unit nrfABCDEFG which is activated in anaerobic conditions.

Cluster n. 247 (of 3 genes) The genes form the plsX-fabHDG transcription unit.

Cluster n. 248 (of 3 genes) tnaCAB is included in the cluster.

Cluster n. 249 (of 3 genes) The acs-yjcHG TU is fully included.

Cluster n. 250 (of 3 genes) It consists of all the three components of MglABC, that is a beta-methylgalactoside transport system, necessary for beta-methylgalactoside transport function.

Cluster n. 251 (of 3 genes) The 3 genes form the glpABC operon which codes for the glycerol-3-phosphate-dehydrogenase, anaerobic.

Cluster n. 252 (of 3 genes) The three genes belong to the same TU: lldPRD.

Cluster n. 253 (of 3 genes) The three genes belong to the same TU: betIBA.

Cluster n. 254 (of 3 genes) cadBA and the gene located upstream are included in this cluster.

Cluster n. 255 (of 3 genes) The genes are part of the integral membrane protein of SoxR-reducing complex

Cluster n. 256 (of 3 genes) The genes lie on the operon glnHPQ and form the glutamine ABC transporter complex.

Cluster n. 257 (of 3 genes) yedX-Y-Z are three consecutive genes.

Cluster n. 258 (of 3 genes) NA

Cluster n. 259 (of 3 genes) Cluster of prophage genes: ybbC is DLP12 prophage, predicted exonuclease; NohA is a Qin prophage, predicted packaging protein, finally, YbcT is a DLP12 prophage; predicted murein endopeptidase.

Cluster n. 260 (of 3 genes) The genes belong to the ygbFT-ygcHIJKL TU.

Cluster n. 261 (of 3 genes) Two genes are part of the ygfXY TU.

Cluster n. 262 (of 2 genes) The rng gene encodes RNAse G and is also involved in RNA turnover. For the other gene no information has been found, but they are closely positioned on the genome.

Cluster n. 263 (of 2 genes) These genes form the nudJ-rluE TU.

Cluster n. 264 (of 2 genes) NA

Cluster n. 265 (of 2 genes) Two out of 3 genes that form the ychE1-yhcAD TU are included in this cluster, for which no functional information is known.

Cluster n. 266 (of 2 genes) The two genes are KpLE2 phage-like elements.

Cluster n. 267 (of 2 genes) The cluster contains the genes yjcP and yjcR. Sequence analysis suggests that yjcR is a member of the membrane fusion protein (MFP) family of membrane transporters whose natural substrate is unknown, instead yjcP is predicted to be an outer membrane factor (OMF) family component of a multidrug efflux system. The clustering suggests that this two genes respond cooperatively to the same drugs.

Cluster n. 268 (of 2 genes) The two genes are part of the same TU. GDP-mannose mannosyl hydrolase (wcaH) is able to hydrolyze both GDP-mannose and GDP-glucose. Its biological role is as yet unknown, though it may participate in the regulation of cell wall biosynthesis and in providing GDP for the synthesis of GDP-fucose. WcaF is believed to be an acetyltransferase involved in colanic acid synthesis and production of the Extracellular Polysaccharide Colanic Acid. This last assumption seems to be confirmed by the discovery of its coexpression with wcaH.

Cluster n. 269 (of 2 genes) NA

Cluster n. 270 (of 2 genes) NA

Cluster n. 271 (of 2 genes) NA

Cluster n. 272 (of 2 genes) NA

Cluster n. 273 (of 2 genes) NA

Cluster n. 274 (of 2 genes) NA

Cluster n. 275 (of 2 genes) NA

Cluster n. 276 (of 2 genes) sgcR is part of the sgcABCEQRX TU, in particular it seems to be the sgc operon transcriptional regulator, while the remaining gene is not part of the same TU, but it is located 30855 bp upstream.

Cluster n. 277 (of 2 genes) The two genes form the lgt-thyA operon.

Cluster n. 278 (of 2 genes) NA

Cluster n. 279 (of 2 genes) The 2 genes are part of the aceBAK transcription unit and are involved in the glyoxylate cycle.

Cluster n. 280 (of 2 genes) NA

Cluster n. 281 (of 2 genes) These two genes belong to the same TU dcyD-yecCS.

Cluster n. 282 (of 2 genes) GadW represses the transcription of the gadAX and gadBC operons. GadX is one of the responsible regulators of the glutamate dependent acid-resistance (AR) system of *E.coli*. The regulated operons can be controlled by other 10 transcription factors. Initially, GadX was characterized as a dual regulator, later as an activator. More recently, it is proposed that it regulates along with GadW (another member of the AraC/XylS family). Once again the clustering process is in agreement with the biological hypothesis.

Cluster n. 283 (of 2 genes) NA

Cluster n. 284 (of 2 genes) The two genes are part of the rfe-rffACDEGHMT-wzxE-wzyE-wzzE, and both are involved in enterobacterial common antigen.

Cluster n. 285 (of 2 genes) NA

Cluster n. 286 (of 2 genes) Based on sequence similarity, YebQ (B1828) may function as a proton-driven drug efflux system and YeaN (B1791) as a proton-driven metabolite uptake system. This cluster suggests that the predicted function of these two proteins should be the same and that they may work cooperatively.

Cluster n. 287 (of 2 genes) NA

Cluster n. 288 (of 2 genes) XylE is a member of the major facilitator superfamily (MFS) of transporter. XylE is a D-xylose/proton symporter. The YqcE protein is an uncharacterized member of the MFS family of transporters and it may function as a proton-driven metabolite uptake system. According to the clustering it seems that the action of XylE is mediated by YqcE.

Cluster n. 289 (of 2 genes) yheK and pshM are part of the gspCDEFGHIJKLMO secretion complex.

Cluster n. 290 (of 2 genes) The two probes are referring to the same gene tatD, a magnesium-requiring DNase.

Cluster n. 291 (of 2 genes) The mhpE and mhpA genes have been identified in the mhpABCDFE TU involved in the utilization of aromatic acids as carbon and energy sources.

Cluster n. 292 (of 2 genes) NA

Cluster n. 293 (of 2 genes) NA

Cluster n. 294 (of 2 genes) NA

Cluster n. 295 (of 2 genes) NA

Cluster n. 296 (of 2 genes) ArgT seems to be involved in lysine biosynthesis and arginine biosynthesis. GltI (ybej) is the periplasmic-binding component of the GltJKL glutamate ABC transporter. An interesting aspect is the fact that gltI was shown to be regulated by the FlhDC flagellar transcriptional regulator, but most of the genes regulated by this transcriptional regulator are in cluster 1.

Cluster n. 297 (of 2 genes) Both IleX and IleY have triplet codon-amino acid adaptor activity.

Cluster n. 298 (of 2 genes) NA

Cluster n. 299 (of 2 genes) ynfH and dmsD are both included in ynfEFGH-dmsD.

Cluster n. 300 (of 2 genes) yhfX and yhfY are neighbors in the genome but belong to two different operons.

Cluster n. 301 (of 2 genes) The two genes are closely related but belong to two different operons.

Cluster n. 302 (of 2 genes) NA

Cluster n. 303 (of 2 genes) These two genes form the pldB-yigL TU.

Cluster n. 304 (of 2 genes) NA

Cluster n. 305 (of 2 genes) NA

Cluster n. 306 (of 2 genes) NA

Cluster n. 307 (of 2 genes) The two genes form the gidAB TU involved in a tRNA modification pathway.

Cluster n. 308 (of 2 genes) To survive in extremely acidic environments, *E.coli* has evolved three acid resistance strategies. One of them is dependent upon external arginine. This system employs the two genes of the cluster: AdiA decarboxylase and the AdiC Arginine.

Cluster n. 309 (of 2 genes) NA

Cluster n. 310 (of 2 genes) The two genes form the operon umuDC and the DNA polymerase V protein complex.

Cluster n. 311 (of 2 genes) hflX and hflK are both included in the yjeFE-amiB-mutL-miaA-hfq-hflXKC TU. Moreover HflX has a GTP-binding/GTPase region, and it may be involved in regulation of HflC and HflK activity. This hypothesis is reinforce by this result.

Cluster n. 312 (of 2 genes) These genes belong to the rpmBG transcription unit.

Cluster n. 313 (of 2 genes) NA

Cluster n. 314 (of 2 genes) NA

Cluster n. 315 (of 2 genes) NA

Cluster n. 316 (of 2 genes) NA

Cluster n. 317 (of 2 genes) Both genes belong to the same TU: rfe-rffACDEGHMT-wzxE-wzyE-wzzE.

Cluster n. 318 (of 2 genes) B1758 is a predicted phosphatidyl transferase, inner membrane protein, while the ydhP gene probably constitutes a monocistronic operon and, according to sequence similarity, it may function as a proton-driven drug or sugar efflux system.

Cluster n. 319 (of 2 genes) NA

Cluster n. 320 (of 2 genes) The two genes form the yceD-rpmF TU.

Cluster n. 321 (of 2 genes) Rpe is involved in the pentose phosphate pathway. The gph gene encodes an enzyme with 2-phosphoglycolate phosphatase activity. This relationship between these two genes suggests that they take part to a common pathway.

Cluster n. 322 (of 2 genes) The two genes belong to the glnALG TU, involved in the synthetase of glutamine.

Cluster n. 323 (of 2 genes) YleA is a isopentenyl-adenosine A37 tRNA methylthiolase MiaB, while YbjF a 23S ribosomal RNA 5-methyluridine methyltransferase.

Cluster n. 324 (of 2 genes) The two genes are closely positioned on the genome.

Cluster n. 325 (of 2 genes) The two genes belong to the same TU ybjC-nfsA-rimK-ybjN.

Cluster n. 326 (of 2 genes) Two of the 3 genes belonging to the ydeRST TU are included in the cluster.

Cluster n. 327 (of 2 genes) NA

Cluster n. 328 (of 2 genes) The yedEF TU is included in this cluster.

Cluster n. 329 (of 2 genes) The guaBA TU is included in this cluster.

Cluster n. 330 (of 2 genes) MukE and MukF are part of the MukB-MukE-MukF complex.

Cluster n. 331 (of 2 genes) Both genes included, napG and napH, are required for efficient electron transfer via NapC to the NapAB complex.

Cluster n. 332 (of 2 genes) aroF and tyrA form the tyrosine operon.

Cluster n. 333 (of 2 genes) NA

Cluster n. 334 (of 2 genes) NA

Cluster n. 335 (of 2 genes) The two genes form the yqeIJ TU.

Cluster n. 336 (of 2 genes) We have here two subunits of the YnjC/YnjD ABC transporter, an ATP-binding component of a predicted metabolite uptake ABC transporter.

Cluster n. 337 (of 2 genes) The two genes form the uspD-yiiS involved in response to stress.

Cluster n. 338 (of 2 genes) The two genes form the yjbQR TU.

Cluster n. 339 (of 2 genes) The two genes belong to yedN-1N-2.

Cluster n. 340 (of 2 genes) Both probes are referring to the same gene, cpxP, involved in resistance to extracytoplasmic stresses. CpxP is a negative regulator of the Cpx response.

Cluster n. 341 (of 2 genes) The two genes belong to the fimAICDFGH TU.

Cluster n. 342 (of 2 genes) The two genes form the obgE-yhbE involved in DNA replication and ribosome assembly.

Cluster n. 343 (of 2 genes) The genes form the relEB transcription unit, inhibiting protein translation by catalyzing cleavage of mRNA.

Cluster n. 344 (of 2 genes) The genes annotated in this cluster are adjacent on the genome.

Cluster n. 345 (of 2 genes) The two genes belong to the tdcABCDEFG TU.

Cluster n. 346 (of 2 genes) NA

Cluster n. 347 (of 2 genes) The TauABC transporter, of which we have the A and B component, belongs to the ABC superfamily, and is believed to be responsible for taurine uptake as a source of sulfur.

Cluster n. 348 (of 2 genes) NA

Cluster n. 349 (of 2 genes) The genes form the rpmIp transcription unit.

Cluster n. 350 (of 2 genes) The genes belong to the rdgB-yggSTUW TU.

Cluster n. 351 (of 2 genes) The genes belong to the coaE-yacFG TU.

Cluster n. 352 (of 2 genes) nurG and murD are part of the same TU: mraZW-ftsLI-murEF-mraY-murD-ftsW-murGC-ddlB-ftsQAZ.

Cluster n. 353 (of 2 genes) SecD/SecF/YajC (the first two included in the cluster) and YidC (cluster 6) are all components of the Sec protein secretion pathway.

Cluster n. 354 (of 2 genes) NA

Cluster n. 355 (of 2 genes) The two genes form the cspC-yobF TU.

Cluster n. 356 (of 2 genes) The two genes form the panCB unit involved in the formation of an enzyme-pantoyl-AMP intermediate.

Cluster n. 357 (of 2 genes) The ArtPMQJI arginine transporter is a member of the ABC transporter superfamily. In this cluster artP and artI are present.

Cluster n. 358 (of 2 genes) The*E.coli* chaperone protein GroEL (Hsp60) and its regulator GroES are necessary for the proper folding of certain proteins. GroEL and GroES are both heat inducible but are also expressed constitutively and are required for growth. This is an example in which TF and BS are recovered.

Cluster n. 359 (of 2 genes) Rph has ribonuclease PH (RNase PH) activity while yjjT is involved in rRNA methylation.

Cluster n. 360 (of 2 genes) The genes belongs to the same TU: yrbBCDEF.

Cluster n. 361 (of 2 genes) The cluster represents the yabPQ TU.

Cluster n. 362 (of 2 genes) The two genes are closely located on the genome.

Cluster n. 363 (of 2 genes) G7121 is a phantom gene located between two genes of the same TU yegRZ, one of them is part of the cluster.

Cluster n. 364 (of 2 genes) It contains 2 of the 3 genes of the operon csgBAC (csgC is missing).

Cluster n. 365 (of 2 genes) Both genes (fepC and fes) are involved in Fe acquisition.

Cluster n. 366 (of 2 genes) The two genes form the hha-ybaJ TU.

Cluster n. 367 (of 2 genes) The two genes belong to the mraZW-ftsLI-murEF-mraY-murD-ftsW-murGC-ddlB-ftsQAZ TU, involved in cell division.

Cluster n. 368 (of 2 genes) Two genes of the operon tolB-pal-ybgF compose this cluster. They also enter into the Colicin S4 Transport System and in the TolA-Pal Cell Envelope Complex, together with the tolR and tolQ pair (in cluster 409).

Cluster n. 369 (of 2 genes) The two genes form the dcuB-fumB TU.

Cluster n. 370 (of 2 genes) NA

Cluster n. 371 (of 2 genes) The two genes belong to the same TU: lsrACDBFG.

Cluster n. 372 (of 2 genes) The two genes belong to the same TU: ydhVWY.

Cluster n. 373 (of 2 genes) NA

Cluster n. 374 (of 2 genes) The two genes are part of the ydjGHIJKL TU.

Cluster n. 375 (of 2 genes) The two genes are one after the other on the genome.

Cluster n. 376 (of 2 genes) The 2 genes form the gapC_1C_2 transcription unit involved in splitting glyceraldehyde 3-phosphate dehydrogenase C.

Cluster n. 377 (of 2 genes) The two genes are part of the same TU, ydjA-selD-topB.

Cluster n. 378 (of 2 genes) The two genes are part of the same TU, fruBKA. FruAB is the fructose PTS permease, while FruK is a homologue of phosphofructokinase.

Cluster n. 379 (of 2 genes) NA

Cluster n. 380 (of 2 genes) The two genes belong to the def-fmt transcription unit.

Cluster n. 381 (of 2 genes) G6553 is a phantom gene just in front of mdtG.

Cluster n. 382 (of 2 genes) RcsD (YojN) belongs to the phosphorelay system RcsC/RcsD/RcsB, and is involved in the regulation of the synthesis of colanic acid capsule and motility, like RcsC .

Cluster n. 383 (of 2 genes) The genes form the yneGH TU.

Cluster n. 384 (of 2 genes) b1458 is involved in DNA recombination and transposition, like b1459.

Cluster n. 385 (of 2 genes) NA

Cluster n. 386 (of 2 genes) NA

Cluster n. 387 (of 2 genes) The genes belong to holB-pabC-tmk-yceG-ycfH operon.

Cluster n. 388 (of 2 genes) The genes form nrdDG. Moreover NrdD reductase is activated by the NrdG activase under anaerobic conditions and is inactivated by oxygen.

Cluster n. 389 (of 2 genes) The two genes are part of the ivbL-ilvBN operon. They form the acetohydroxybutanoate synthase/acetolactate synthase complex and code for one of the enzymes involved in the isoleucine and valine biosynthesis.

Cluster n. 390 (of 2 genes) The two probes are referring to ycgH-1 and ycgH-2.

Cluster n. 391 (of 2 genes) The two genes fumA and fumC are separated by SoxS.

Cluster n. 392 (of 2 genes) The genes are part of the rzpR-ydaSTUVW TU.

Cluster n. 393 (of 2 genes) The 2 genes are part of the glcDEFGBA transcription unit. Glycolate oxidase catalyzes the first step in the utilization of glycolate as the sole source of carbon.

Cluster n. 394 (of 2 genes) The genes ptsH and gapA have no common annotation.

Cluster n. 395 (of 2 genes) The genes form the yehTU TU.

Cluster n. 396 (of 2 genes) The genes are part of the ytfMNP TU.

Cluster n. 397 (of 2 genes) NA

Cluster n. 398 (of 2 genes) NA

Cluster n. 399 (of 2 genes) These two genes are part of the ygcNOP transcription unit involved in oxidoreductase (ygcP is missing).

Cluster n. 400 (of 2 genes) The genes belong to the paaABCDEFGHIJK TU, a phenylacetate-CoA oxygenase involved in phenylacetate catabolism.

Cluster n. 401 (of 2 genes) The two genes form the cydAB transcription unit.

Cluster n. 402 (of 2 genes) glnV and X are both part of the same operon metT-leuW-glnUW-metU-glnVX.

Cluster n. 403 (of 2 genes) The genes are part of lacZYA operon.

Cluster n. 404 (of 2 genes) The two genes form the dadAX TU.

Cluster n. 405 (of 2 genes) The two genes form the hepA-rluA TU.

Cluster n. 406 (of 2 genes) yddB belongs to the yddAB TU, while the upstream gene ppqL belongs to a monocystic operon.

Cluster n. 407 (of 2 genes) NA

Cluster n. 408 (of 2 genes) NA

Cluster n. 409 (of 2 genes) The pair tolR and tolQ enters into the ybgC-tolQRA transcription unit and in the TolA-Pal Cell Envelope Complex, jointly with the genes of cluster 368.

Cluster n. 410 (of 2 genes) The genes are part of yddJKL TU.

Cluster n. 411 (of 2 genes) The two genes are one after the other on the genome.

Cluster n. 412 (of 2 genes) tyrT and tyrV are part of tyrTV-tpr.

Cluster n. 413 (of 2 genes) NA

Cluster n. 414 (of 2 genes) OppABCDF is an ATP-dependent oligopeptide transporter. In this cluster oppA and oppB are included, while the others are identified in cluster 240.

Cluster n. 415 (of 2 genes) The 2 genes form the ibpAB transcription unit involved in response to oxidative stress.

Cluster n. 416 (of 2 genes) NA

Cluster n. 417 (of 2 genes) NA

Cluster n. 418 (of 2 genes) NA

Cluster n. 419 (of 2 genes) The two genes form the pgk-fbaA transcription unit.

Cluster n. 420 (of 2 genes) These genes belong to the yebYZ-yobA TU.

Cluster n. 421 (of 2 genes) The genes form the paaXY TU, in particular PaaX is a negative regulatory protein that participates in controlling transcriptional regulation of divergent paa catabolic operons involved in phenylacetic acid degradation, and paaY gene is part of the gene cluster required for growth on phenylacetate as the sole source of carbon in *E.coli.*

Cluster n. 422 (of 2 genes) The cluster represents the alaWX TU.

Cluster n. 423 (of 2 genes) NA

Cluster n. 424 (of 2 genes) The two genes are part of the hybOABCDEFG transcription unit.

Cluster n. 425 (of 2 genes) The two genes form the mdtIJ transcription unit.

Cluster n. 426 (of 2 genes) The two genes belong to the yqaAB TU.

Cluster n. 427 (of 2 genes) The two genes form the yeaR-yoaG TU.

Cluster n. 428 (of 2 genes) HybOABCDEFG TU is represented here by two of its components.

Cluster n. 429 (of 2 genes) The yciBC TU belongs to this cluster.

Cluster n. 430 (of 2 genes) The two genes are part of the sapABCDF transcription unit and form the peptide uptake ABC transporter.

Cluster n. 431 (of 2 genes) The genes form the smf-smg TU.

Cluster n. 432 (of 2 genes) The genes belong to the same TU: fabR-yijD.

3 *S.cerevisiae* correlation clusters

The protein complexes subunits considered are those of MIPS, with some additions from SGD; the metabolic pathways are drawn from SGD. Using GO, for every cluster with more than one gene annotated an enrichment was done in order to asses the significant BP, MF or CC categories. The significance was calculated using an hypergeometric distribution with a multiplicity correction of the p.value (via a bootstrapping method).

The main functional annotations retrieved by the clustering procedure are related to chromatin modification, mRNA metabolic process, ribosome biogenesis and assembly, protein transport, proteolysis, regulation of transcription, RNA export from nucleus mitosis, response to DNA damage stimulus, and DNA replication, showing how the best reconstructed processes are those involved in synthesis of protein at the early stages. Metabolic functions seldom emerge. Instead, often times genes included in the same cluster co-localized in the nucleus (2, 4 and many more), mitochondrion (3, 5, 8) and cytoplasm (1, 10, etc.). The most significant clusters in this sense are (by far) the largest ones. In particular, mitochondrial clusters are very compact also for metabolic functions such as the oxidative phosphorylation (as already described in the paper).

3.1 Functional annotation of the clusters

Cluster n. 1 (of 138 genes) The vast majority of the genes belonging to the cluster is involved in the production of ribonucleoproteins and is located in the cytoplasm. In particular all the 49 genes that are structural constituents of the cytoplasmic ribosomal small subunit and 70, out of the 72, forming the cytoplasmic ribosomal large subunit, are present here. In addition, the EGD1 and EGD2 genes, encoding the yeast nascent polypeptide-associated complex (NAC), are included in the cluster, together with genes involved in rRNA processing and translation initiation, confirming the observation that the cluster is associated with cytoplasmic ribosomes.

Cluster n. 2 (of 120 genes) This cluster is mainly associated with the nuclear compartment. It contains rRNA splicing (10/14), Chaperonine containing T-complex TRiC (TCP RING Complex) (7/8), RNA polymerase I (7/14), RNA polymerase III (7/13), eIF2B (5/5), Nop56p/Nop1p complex (3/3) and Exosome complex (6/7), so basically genes related to mRNA processing and transcription regulation from polymerases I and III. Purine and pyrimidine metabolic pathways are also represented by 11 genes.

Cluster n. 3 (of 70 genes) Most of the genes belonging to the cluster are localized in the mitochondria and concur to the formation of the mitochondrial ribosomal small subunit (23/29) and the mitochondrial ribosomal large subunit (33/44). Also the remaining genes are localized in the same compartment with high significance: 6 are involved in protein targeting to mitochondria (Tim22p-complex (2/5) and Tim9p/Tim10p-complex (2/2)).

Cluster n. 4 (of 36 genes) Like in cluster 2, there is a predominant presence of genes located in the nucleus and involved in mRNA processing, such as RNA polymerase I (4/14), II (6/13) and III (4/13). Other genes are related to tubulin folding (Gim complexes (4/5)) and RNA splicing (snRNP U2 and U6). Purine and pyrimidine metabolism is represented by 7 genes. Another group of genes belongs to the translocon family of complexes (signal recognition particle 3/6, signal peptidase 1/3, and Sec61 complex 1/2).

Cluster n. 5 (of 36 genes) Of the 36 genes, 34 are mitochondrial and involved in oxidative phosphorylation: Succinate dehydrogenase complex (complex II) (4/4) Cytochrome bc1 complex (Ubiquinol-cytochrome c reductase complex III) (8/9), Cytochrome c oxidase (complex IV) (8/8), and F0/F1 ATP synthase (complex V) (14/14). Of the two remaining genes, one is HAP4, i.e., the principal activator of the Hap2p/3p/4p/5p CCAAT-binding complex, the major transcription factor of the respiratory chain complexes [2].

Cluster n. 6 (of 34 genes) Most of the genes are involved in proteolysis (28 out of the 34 belonging to this cluster are subunits of the proteasome macrocomplex). Of the 14 genes forming the 20S proteasome, 12 are in the cluster, in addition to 13 out of 18 of the 19/22S regulator complex. One of the remaining genes is part of the SCF complex which catalyzes ubiquitilation of proteins to be degraded in the proteasome.

Cluster n. 7 (of 31 genes) Nuclear envelope is the overrepresented compartment in the cluster, as it contains part of the nuclear pore complex (NPC) (5/13), with other genes related to RNA export from nucleus and rRNA processing. Other four genes are involved in purine and pyrimidine metabolism. For the categories of mRNA processing and translation initiation, this cluster seems to complement cluster 2. The following complexes are in fact shared between the two: RNA Polymerase I (2/14), III (2/13), rRNA spicing (1/14), eIF2 (1/3), eIF3 (3/5), eIF4E/eIF4G/Pab1p (1/3) and Exosome (1/7).

Cluster n. 8 (of 26 genes) Phosphofructokinase (2/2) and Pyruvate dehydrogenase (2/5), two of the irreversible steps of the Glycolysis pathway, are co-clustered here, mostly with genes from the mitochondrial outer and inner membrane translocases (TOM: 4/7; TIM: 2/10) and a few mitochondrial ribosomal subunits.

Cluster n. 9 (of 24 genes) The genes colocalize in the nucleus and are related to RNA splicing and endoribonuclease activity. In particular 4/8 subunits of the RNase complex.

Cluster n. 10 (of 24 genes) The genes belonging to the cluster are involved in regulation of transcription, mitotic cell cycle and protein targeting.

Cluster n. 11 (of 20 genes) The majority of the 20 genes that belong to this cluster is involved in transcription, including genes belonging to RNA polymerase I-II, pre mRNA 3'-end processing factors CFII and PFI, RSC complex (remodeling of the structure of chromatin), SAGA complex and several transcription factors subunits from TFIIA, TFIIF, TFIIH, TAFIIs, and TFIIIB. There are also a few elongation factors (eEF1, eIF4F).

Cluster n. 12 (of 18 genes) Five genes are involved in phosphate metabolic processes, three in hydrolase activity, acting on ester bonds, and two in oxidative phosphorylation. The starch and sucrose metabolic pathway is represented here by 3 genes out of 7, and cAMP-dependent protein kinase is identified by 2 genes out of 3.

Cluster n. 13 (of 16 genes) The relevant function is rRNA processing and the overrepresented compartment is the nucleus.

Cluster n. 14 (of 15 genes) This cluster is related to DNA replication. The replication factor A complex is fully included, in addition to DNA polymerase alpha (3/4), delta (1/3), pre-replication complex (1/4) and sister chromatid cohesion complex (2/6).

Cluster n. 15 (of 11 genes) The genes are mainly located in the endoplasmic reticulum membrane. Five (out of 9) subunits of the Oligosaccharyltransferase co-cluster with 3 (out of 13) genes of the vacuolar H+-ATPase and 1 (out of 3) of the signal peptidase. The Oligosaccharyltransferase participates in the N-Glycan biosynthesis pathway.

Cluster n. 16 (of 11 genes) Two basic mitochondrial multienzyme complexes, 2-oxoglutarate dehydrogenase (2/3) and Pyruvate dehydrogenase (2/5), are co-clustered with actin-associated proteins (2/4).

Cluster n. 17 (of 10 genes) This cluster is related to chromatin modification and remodeling (histone deacetylase and acetyltransferase, various cleavage and splicing factors).

Cluster n. 18 (of 10 genes) Genes are involved in nuclear mRNA splicing, via spliceosome, and DNA repair.

Cluster n. 19 (of 10 genes) Genes are mainly related to mRNA processing.

Cluster n. 20 (of 10 genes) A basic function in the cluster is RNA export from nucleus, containing 3 out of the 6 genes forming NUP84 complex.

Cluster n. 21 (of 10 genes) Seven genes are involved in amino acid metabolic processes and two in glutamate metabolism. They form 4 enzymatic complexes, 3 synthases (Arginine-specific carbamoylphosphate synthase [2/2], Anthranilate synthase [2/2] and Acetolactate synthase [1/2]) and a reductase (sulfite reductase complex [2/2]).

Cluster n. 22 (of 9 genes) Protein amino acid glycosylation and protein targeting are overrepresented functions in the cluster, including TOM - transport across the outer membrane (3/7), located in the mitochondria, and oligosaccharyltransferase (3/9), located in the ER-lumen.

Cluster n. 23 (of 9 genes) The genes are involved in protein targeting and chromatin modification.

Cluster n. 24 (of 8 genes) The two main functions retrieved in the cluster are regulation of transcription from RNA polymerase II promoter and sporulation.

Cluster n. 25 (of 8 genes) Genes are related to mitotic cell cycle, regulation of transcription from RNA polymerase II promoter and to mRNA processing.

Cluster n. 26 (of 8 genes) Genes are located in the nuclear chromosome and mainly involved in negative regulation of transcription and mitotic cell cycle.

Cluster n. 27 (of 8 genes) Three genes are involved in RNA splicing and two in the G1-specific transcription in mitotic cell cycle.

Cluster n. 28 (of 8 genes) NA

Cluster n. 29 (of 7 genes) Five genes are involved in proteolysis.

Cluster n. 30 (of 7 genes) The functions belonging to the cluster are: secretory pathway and carbohydrate metabolic process, including 2 genes belonging to Glycolysis/Gluconeogenesis (Enolase isoenzymes 2/2). This cluster contains the last two genes forming the cytoplasmic ribosomal large subunit missing from cluster 1.

Cluster n. 31 (of 7 genes) Genes are mainly related to lipid metabolic process and kinase regulator activity.

Cluster n. 32 (of 6 genes) Most of the genes are located in the nucleus and are related to transcription from RNA polymerase I promoter (3 genes belong to the Core Factor (3/4)).

Cluster n. 33 (of 6 genes) NA

Cluster n. 34 (of 6 genes) NA

Cluster n. 35 (of 6 genes) All genes are involved in RNA processing and replication complexes.

Cluster n. 36 (of 6 genes) The features of this cluster are similar to the previous one. Two genes are part of the replication complex (2/19).

Cluster n. 37 (of 6 genes) Four genes are related to cytoskeleton organization and biogenesis, and they co-localize in the cytoskeleton. The complexes included in the cluster are: Actin-associated proteins (2/4), Arp2p/Arp3p complex (3/6).

Cluster n. 38 (of 6 genes) NA

Cluster n. 39 (of 6 genes) Three genes are related to proteolysis.

Cluster n. 40 (of 6 genes) Five out of the six genes belonging to the cluster are related to helicase activity. Four of them participate in the pre-replication complex (4/14).

Cluster n. 41 (of 5 genes) NA

Cluster n. 42 (of 5 genes) Two nuclear envelope elements are co-clustered with 3 transferase enzymatic genes.

Cluster n. 43 (of 5 genes) Four genes are localized in the integral membrane and are related to nuclear organization and biogenesis as we can also deduce by the complex corresponding to the cluster: nuclear pore complex (NPC) (2/13).

Cluster n. 44 (of 5 genes) Two genes are belonging to the pheromone response pathway.

Cluster n. 45 (of 5 genes) All genes are involved in transcriptional processes.

Cluster n. 46 (of 5 genes) The five genes are involved in regulation of progression through cell cycle and mitotic spindle elongation, in fact two concur to the formation of the anaphase promoting complex (2/11).

Cluster n. 47 (of 4 genes) NA

Cluster n. 48 (of 4 genes) Three genes are involved in nuclear mRNA splicing, via spliceosome and two of them belong to the mRNA splicing complex.

Cluster n. 49 (of 4 genes) Nuclear organization and biogenesis is represented by 3 genes, 2 part of the nuclear pore complex.

Cluster n. 50 (of 4 genes) NA

Cluster n. 51 (of 4 genes) Three genes are related to mRNA export from nucleus.

Cluster n. 52 (of 4 genes) NA

Cluster n. 53 (of 4 genes) NA

Cluster n. 54 (of 4 genes) All the genes are located in the condensed nuclear chromosome and two of them belong to the sister chromatid cohesion complex (2/6).

Cluster n. 55 (of 4 genes) Most of the genes are involved in chromatin assembly or disassembly.

Cluster n. 56 (of 4 genes) Almost all the genes are related to negative regulation of transcription through chromatin silencing.

Cluster n. 57 (of 4 genes) All the genes are located in the mitochondrial matrix and are part of the mitochondrial ribosomal large subunit (4/44) (see cluster 3).

Cluster n. 58 (of 4 genes) NA

Cluster n. 59 (of 4 genes) NA

Cluster n. 60 (of 4 genes) Genes are involved in protein polyubiquitination and carbohydrate metabolic process.

Cluster n. 61 (of 4 genes) NA

Cluster n. 62 (of 4 genes) The four genes are related to mitotic cell cycle and cytoskeleton organization and biogenesis.

Cluster n. 63 (of 4 genes) NA

Cluster n. 64 (of 4 genes) NA

Cluster n. 65 (of 4 genes) NA

Cluster n. 66 (of 4 genes) NA

Cluster n. 67 (of 4 genes) The genes are related to helicase activity.

Cluster n. 68 (of 4 genes) Three genes are related to transcription initiation.

Cluster n. 69 (of 3 genes) Two genes are involved in DNA replication.

Cluster n. 70 (of 3 genes) NA

Cluster n. 71 (of 3 genes) NA

Cluster n. 72 (of 3 genes) Two genes are involved in the M phase of mitotic cell cycle.

Cluster n. 73 (of 3 genes) Two genes are part of the ribonucleoside-diphosphate reductase complex (2/4).

Cluster n. 74 (of 3 genes) NA

Cluster n. 75 (of 3 genes) NA

Cluster n. 76 (of 3 genes) NA

Cluster n. 77 (of 3 genes) Two genes are involved in the fatty acid biosynthetic process and form the cytoplasmic fatty acid synthetase complex (2/2).

Cluster n. 78 (of 3 genes) NA

Cluster n. 79 (of 3 genes) The annotated genes are located in the nuclear chromosome and are involved in meiosis.

Cluster n. 80 (of 3 genes) Transcription regulation involves two genes of the cluster (part of the nucleotide-excision repair machinery).

Cluster n. 81 (of 3 genes) NA

Cluster n. 82 (of 3 genes) NA

Cluster n. 83 (of 3 genes) NA

Cluster n. 84 (of 3 genes) Two out of the three genes belonging to the cluster are related to mitotic cell cycle, and colocalize in the nuclear chromosome.

Cluster n. 85 (of 3 genes) Negative regulation of transcription involves two genes of the cluster.

Cluster n. 86 (of 3 genes) Two genes are involved in protein amino acid acetylation.

Cluster n. 87 (of 3 genes) NA

Cluster n. 89 (of 3 genes) All the genes are related to protein catabolic process located in the proteasome complex. In particular two of them are belonging to the 19/22S regulator (2/18).

Cluster n. 90 (of 3 genes) NA

Cluster n. 91 (of 3 genes) The three genes are involved in double-strand break repair, transcription initiation and mRNA polyadenylation.

Cluster n. 92 (of 3 genes) The genes are involved in chromatin remodeling and transcription activation.

Cluster n. 93 (of 3 genes) NA

Cluster n. 94 (of 3 genes) Two genes are involved in nuclear mRNA splicing, via spliceosome and colocalize within snRNP U2.

Cluster n. 95 (of 3 genes) NA

Cluster n. 96 (of 3 genes) NA

Cluster n. 97 (of 3 genes) NA

Cluster n. 98 (of 3 genes) Two genes, part of the mRNA splicing complex (2/35), are involved in RNA splicing factor activity, transesterification mechanism, while the remaining one in MAPKKK cascade.

Cluster n. 99 (of 3 genes) NA

Cluster n. 100 (of 3 genes) The genes form the glycine decarboxylase complex (3/4).

Cluster n. 101 (of 3 genes) All the genes are belonging to the chromatin remodeling complex.

Cluster n. 102 (of 3 genes) NA

Cluster n. 103 (of 3 genes) The cluster is associated with mRNA processing.

Cluster n. 104 (of 3 genes) Two genes are involved in DNA-dependent DNA replication.

Cluster n. 105 (of 3 genes) NA

Cluster n. 106 (of 3 genes) NA

Cluster n. 107 (of 3 genes) NA

Cluster n. 108 (of 3 genes) NA

Cluster n. 109 (of 3 genes) NA

Cluster n. 110 (of 3 genes) Two genes are related to negative regulation of transcription from RNA polymerase II promoter and are part of the srb10p complex (2/4).

Cluster n. 111 (of 3 genes) NA

Cluster n. 112 (of 3 genes) NA

Cluster n. 113 (of 3 genes) NA

Cluster n. 114 (of 3 genes) Two genes colocalize in the nuclear envelope.

Cluster n. 115 (of 3 genes) NA

Cluster n. 116 (of 3 genes) NA

Cluster n. 117 (of 2 genes) Both genes are involved in golgi vesicle transport.

Cluster n. 118 (of 2 genes) NA

Cluster n. 119 (of 2 genes) The two genes are involved in protein targeting to mitochondrion and transcription termination.

Cluster n. 120 (of 2 genes) NA

Cluster n. 121 (of 2 genes) NA

Cluster n. 122 (of 2 genes) NA

Cluster n. 123 (of 2 genes) The two genes are involved in nuclear mRNA splicing, via spliceosome and DNA binding.

Cluster n. 124 (of 2 genes) Two genes are involved in adaptation to pheromone during conjugation with cellular fusion and meiosis.

Cluster n. 125 (of 2 genes) Protein complex assembly and tRNA processing are the two functions associated to the cluster.

Cluster n. 126 (of 2 genes) The pair of genes belonging to the cluster are involved in translational elongation and RNA polymerase II transcription factor activity.

Cluster n. 127 (of 2 genes) Both genes are involved in chromatin modification.

Cluster n. 128 (of 2 genes) One gene is involved in transcription elongation regulator activity and the other one is part of the mRNA cap complex.

Cluster n. 129 (of 2 genes) NA

Cluster n. 130 (of 2 genes) NA

Cluster n. 131 (of 2 genes) One gene is related to transcription initiation while the other one in mRNA polyadenylation.

Cluster n. 132 (of 2 genes) Both genes are involved in mRNA splicing.

Cluster n. 133 (of 2 genes) NA

Cluster n. 134 (of 2 genes) NA

Cluster n. 135 (of 2 genes) The two genes are related to acyltransferase activity and oxidoreductase activity.

Cluster n. 136 (of 2 genes) Functions related to the cluster are: cell wall mannoprotein biosynthetic process and regulation of progression through cell cycle.

Cluster n. 137 (of 2 genes) The genes are involved in G2/M transition of mitotic cell cycle and meiotic mismatch repair.

Cluster n. 138 (of 2 genes) NA

Cluster n. 139 (of 2 genes) Both genes are part of the transcription elongation factor complex.

Cluster n. 140 (of 2 genes) NA

Cluster n. 141 (of 2 genes) NA

Cluster n. 142 (of 2 genes) NA

Cluster n. 143 (of 2 genes) Both genes are involved in cellular ion homeostasis.

Cluster n. 144 (of 2 genes) NA

Cluster n. 145 (of 2 genes) Both genes are involved in glycogen metabolic process and are part of the cyclin dependent protein kinase holoenzyme.

Cluster n. 146 (of 2 genes) The two genes are related to mRNA cleavage and export from nucleus.

Cluster n. 147 (of 2 genes) The genes are belonging to regulation of transcription from RNA polymerase II promoter.

Cluster n. 148 (of 2 genes) Both genes are located in the nuclear envelope and are involved in transport of protein and mRNA into and out the nucleus.

Cluster n. 149 (of 2 genes) NA

Cluster n. 150 (of 2 genes) The two genes are involved in nucleoside-triphosphatase activity and clathrin binding.

Cluster n. 151 (of 2 genes) NA

Cluster n. 152 (of 2 genes) Both genes belong to the chromatin remodeling complex.

Cluster n. 153 (of 2 genes) The genes are related to nucleotide binding and RNA polymerase II transcription factor activity.

Cluster n. 154 (of 2 genes) Both genes are related to transcription.

Cluster n. 155 (of 2 genes) NA

Cluster n. 156 (of 2 genes) Both genes are involved in protein ubiquitination.

Cluster n. 157 (of 2 genes) NA

Cluster n. 158 (of 2 genes) Both genes are involved in protein amino acid dephosphorylation.

Cluster n. 159 (of 2 genes) The genes are related to chromatin silencing and RNA polymerase II transcription factor activity.

References

[1] A. de la Fuente, N. Bing, I. Hoeschele, and P. Mendes. Discovery of meaningful associations in genomic data using partial correlation coefficients. *Bioinformatics*, 20(18):3565–3574, 2004.

[2] S. L. Forsburg and L. Guarente. Identification and characterization of hap4: a third component of the ccaat-bound hap2/hap3 heteromer. *Genes Dev*, 3(8):1166–1178, Aug 1989.

[3] X. Guo, R. Liu, C. D. Shriver, H. Hu, and M. N. Liebman. Assessing semantic similarity measures for the characterization of human regulatory pathways. *Bioinformatics*, 22(8):967–973, Apr 2006.

[4] T. Ikebe, S. Iyoda, and K. Kutsukake. Structure and expression of the flia operon of salmonella typhimurium. *Microbiology*, 145 (Pt 6):1389–1396, 1999.

[5] D. Lin. An information-theoretic definition of similarity. In *Proceedings of the 15-th International Conference on Machine Learning*, pages 296–304, 1998.

[6] F. Poli, C. Romagnoli, G. Dall'Olio, and M. P. Fasulo. Effects of spermine and spermidine on cell division and wall morphogenesis in saccharomyces cerevisiae. *Microbios*, 73(297):261–267, 1993.

[7] J. Schäfer and K. Strimmer. An empirical Bayes approach to inferring large-scale gene association networks. *Bioinformatics*, 21(6):754–764, 2005.

[8] N. Soranzo, G. Bianconi, and C. Altafini. Comparing relevance network algorithms for reverse engineering of large scale gene regulatory networks: synthetic vs real data. *Bioinformatics*, 23:1640–1647, 2007.

[9] J. D. Storey and R. Tibshirani. Statistical significance for genome-wide studies. *PNAS*, 100:9440–9445, 2003.
