# Supplementary material for: Origin of Co-Expression Patterns in E.coli and S.cerevisiae Emerging from Reverse Engineering Algorithms
Source: PLoS One. 2008 Aug 20;3(8):e2981. doi: 10.1371/journal.pone.0002981 (PMC2500178; doi:10.1371/journal.pone.0002981)
Supplement: Supplementary Notes S2 — (0.07 MB PDF) [file pone.0002981.s002.pdf]

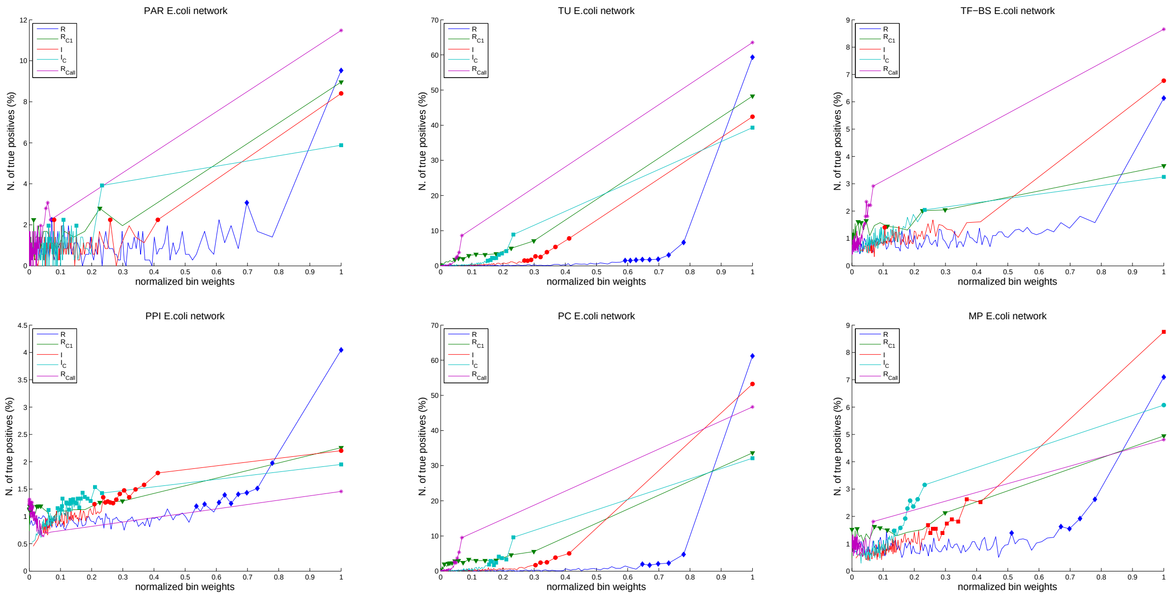

Figure S2: **True edges distribution for *E.coli* networks**. The histograms show the percentage of true edges for each of the “physical” networks described in Table 1(a) of the paper in each of the 100 bins in which the values of the similarity matrix (corresponding to calculated edges weights) is subdivided for the 5 different reconstruction algorithms described in this Supplementary Notes. The binning is according to the inferred edges weights, each bin contains 94373 edges, and the bin weights (taken as the median of the weights of the edges in each bin) are normalized to 1. Overrepresentation towards the heaviest weighted edges is clearly visible for all the reconstruction algorithms and for all 6 physical networks analyzed. A randomization of the edges of any of the physical networks with any of the algorithms (not shown) yields a uniform distribution of the true edges in the bins, detecting a value of $\sim 1\%$ on each bin. In all histograms, bins having a marker correspond to a q.value < 0.05 (see Supplementary Notes S1).
