# Supplementary material for: Origin of Co-Expression Patterns in E.coli and S.cerevisiae Emerging from Reverse Engineering Algorithms
Source: PLoS One. 2008 Aug 20;3(8):e2981. doi: 10.1371/journal.pone.0002981 (PMC2500178; doi:10.1371/journal.pone.0002981)
Supplement: Supplementary Notes S3 — (0.11 MB PDF) [file pone.0002981.s003.pdf]

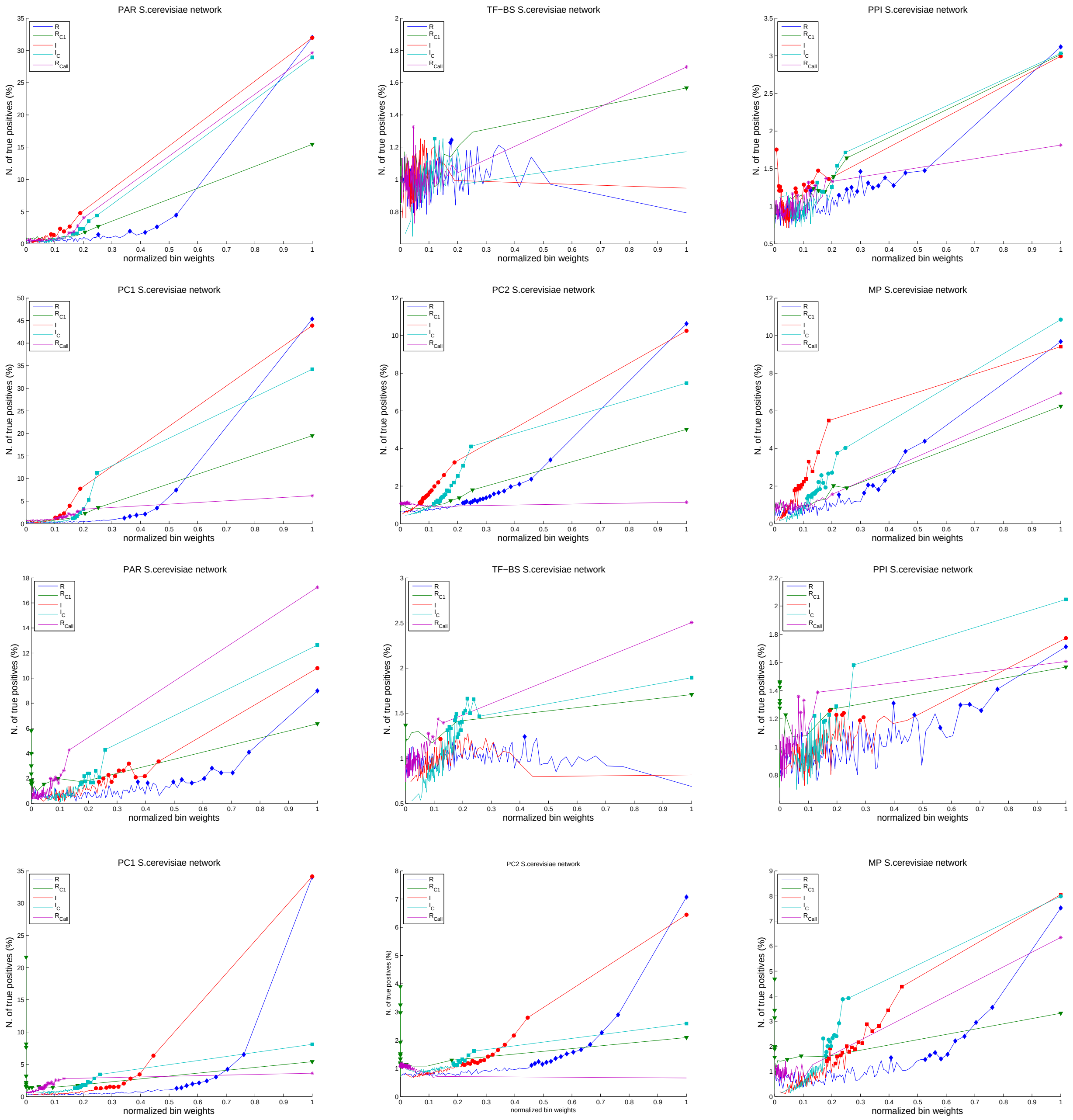

Figure S3: **True edges distribution for the *S.cerevisiae* networks**. The histograms show the percentage of true edges for the 6 "physical" networks described in Table 1(b) of the paper in each of the 100 bins in which the values of the similarity matrix (corresponding to calculated edges weights) is subdivided for the 5 different reconstruction algorithms described in this Supplementary Notes. The binning is according to the inferred edges weights, each bin contains 192355 edges, and the bin weights (taken as the median of the weights of the edges in each bin) are normalized to 1. In the histograms, a marker is plotted in correspondence of the values considered statistically significant ($q.value < 0.05$, see the Supplementary Notes S1). The first two rows are for the cDNA dataset, the last two for the Affymetrix dataset. Overrepresentation towards the heaviest weighted edges is clearly visible for all the reconstruction algorithms on 4 out the 6 physical networks analyzed (both datasets are in agreement). The histograms for the PPI are only weakly skewed, however all top bins for this reconstruction are labeled as significant ($q.value < 0.05$). The only physical network showing an approximately uniform distribution is the TF-BS network, which to a large extent has no statistical significance ($q.value \geqslant 0.05$ also in some of the top bins).
