# Supplementary material for: Origin of Co-Expression Patterns in E.coli and S.cerevisiae Emerging from Reverse Engineering Algorithms
Source: PLoS One. 2008 Aug 20;3(8):e2981. doi: 10.1371/journal.pone.0002981 (PMC2500178; doi:10.1371/journal.pone.0002981)
Supplement: Supplementary Notes S4 — (0.11 MB PDF) [file pone.0002981.s004.pdf]

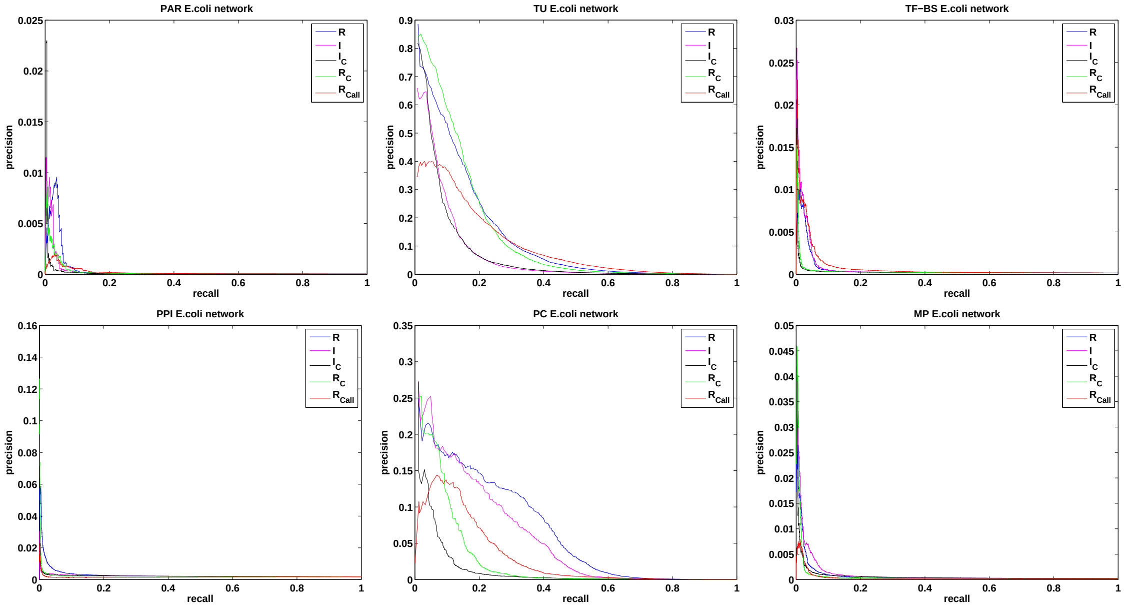

Figure S4: **Precision vs recall curves for *E.coli* networks**. Precision versus recall curves of each similarity matrix for the six networks.
