# Supplementary material for: Origin of Co-Expression Patterns in E.coli and S.cerevisiae Emerging from Reverse Engineering Algorithms
Source: PLoS One. 2008 Aug 20;3(8):e2981. doi: 10.1371/journal.pone.0002981 (PMC2500178; doi:10.1371/journal.pone.0002981)
Supplement: Supplementary Notes S5 — (0.24 MB PDF) [file pone.0002981.s005.pdf]

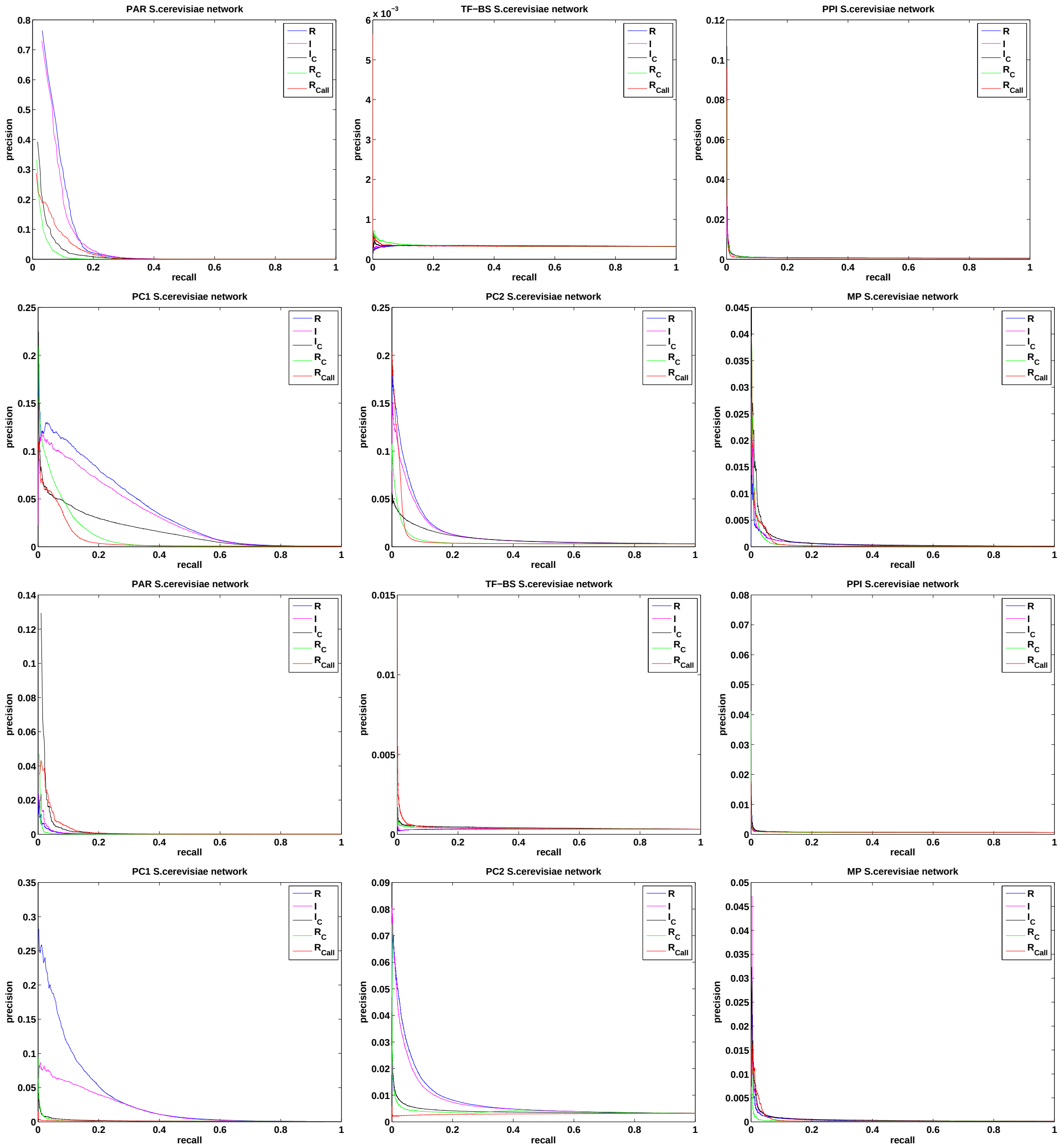

Figure S5: **Precision vs recall curves for *S.cerevisiae* networks**. Precision versus recall curves of each similarity matrix for the six networks. The first two rows are for the cDNA dataset, the last two for the Affymetrix dataset.
