# Supplementary material for: Origin of Co-Expression Patterns in E.coli and S.cerevisiae Emerging from Reverse Engineering Algorithms
Source: PLoS One. 2008 Aug 20;3(8):e2981. doi: 10.1371/journal.pone.0002981 (PMC2500178; doi:10.1371/journal.pone.0002981)
Supplement: Supplementary Notes S6 — (0.04 MB PDF) [file pone.0002981.s006.pdf]

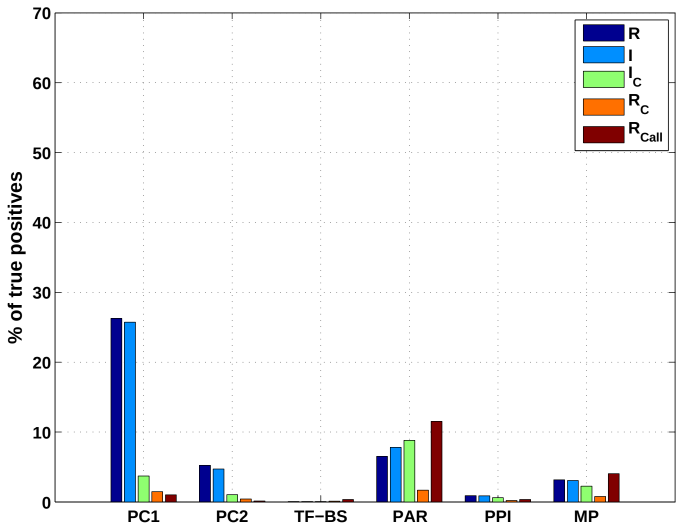

Figure S6: **Overrepresented networks in the top bin of Fig. 1(d) for cDNA and Affymetrix datasets in *S.cerevisiae* (common edges).** The histograms represent the percentage of correct interactions in the top bin (see Fig. 1(d) of the paper) common to both datasets (cDNA and Affymetrix) in *S.cerevisiae*. Most of the information contained in the Affymetrix dataset is retained in the cDNA dataset. This result confirms the observation reported in Fig. S7 where the cDNA collection seems to have a higher content of perturbations. In addition it provides key evidence that certain gene-gene interactions are significant regardless of the specific perturbation applied.
