# Supplementary material for: Origin of Co-Expression Patterns in E.coli and S.cerevisiae Emerging from Reverse Engineering Algorithms
Source: PLoS One. 2008 Aug 20;3(8):e2981. doi: 10.1371/journal.pone.0002981 (PMC2500178; doi:10.1371/journal.pone.0002981)
Supplement: Supplementary Notes S7 — (0.05 MB PDF) [file pone.0002981.s007.pdf]

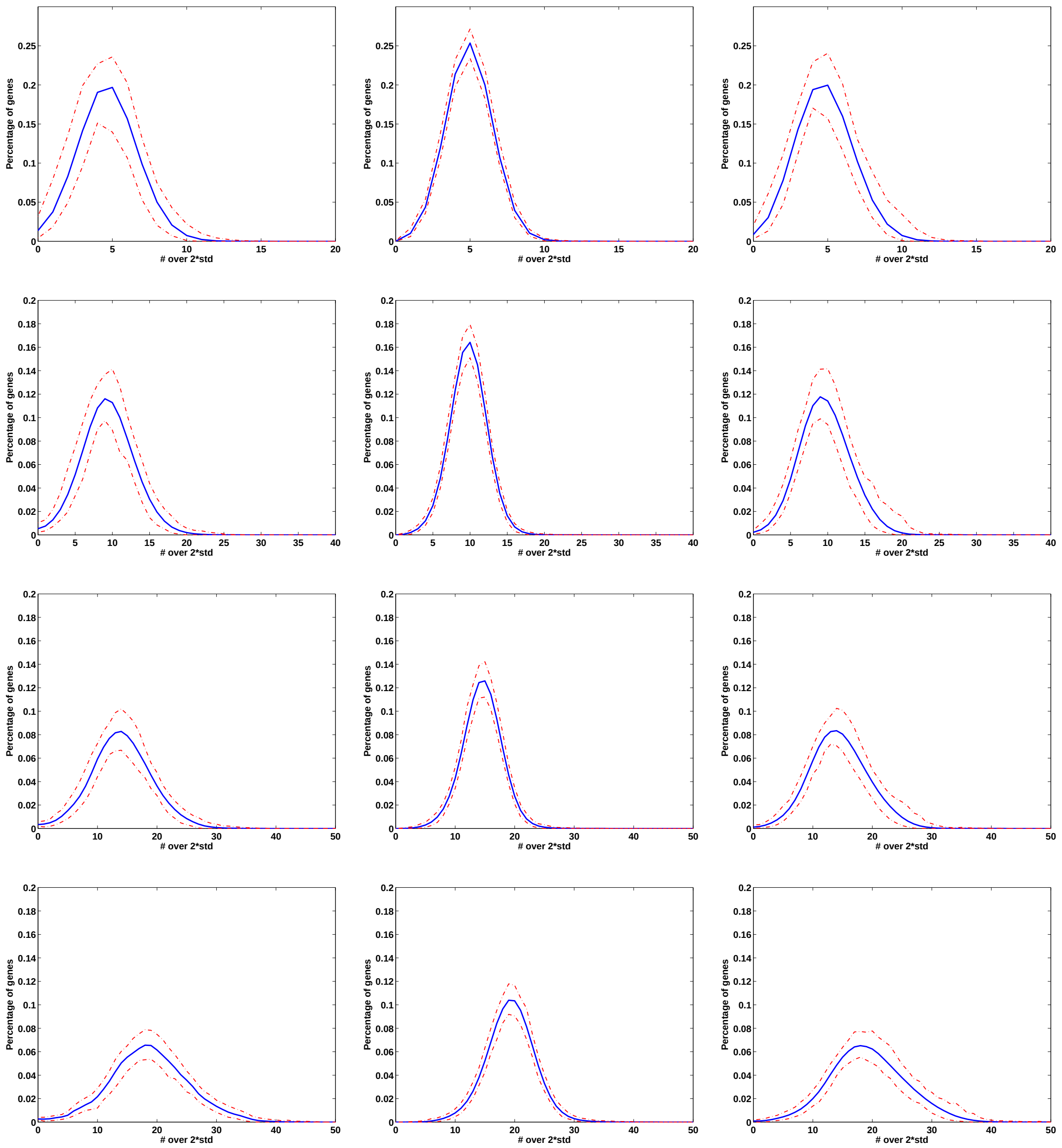

Figure S7: **Datasets variability.** A bootstrapping method is used to estimate the perturbation content of the datasets. Normalization is performed in order to have the same expression distribution for each experiment in a dataset. The statistic estimates for each gene the number of experiments in which the expression value is an outlier of a confidence interval twice the standard deviation. The statistic computation is repeated 1000 times, each time selecting a different sub-groups of samples (from the upper row to the bottom one, 100, 200, 300 and 400 samples are randomly chosen). The resulting averaged distribution curves are represented in blue and are surrounded by the maximum and minimum calculated values (dashed red lines), for *E.coli* (left) and *S.cerevisiae* (cDNA (middle) and Affymetrix (right) datasets). If the Affymetrix datasets are characterized by a similar distribution, for the cDNA dataset the degree of perturbation seems to be higher (i.e. peaks are higher).
