# Supplementary material for: Origin of Co-Expression Patterns in E.coli and S.cerevisiae Emerging from Reverse Engineering Algorithms
Source: PLoS One. 2008 Aug 20;3(8):e2981. doi: 10.1371/journal.pone.0002981 (PMC2500178; doi:10.1371/journal.pone.0002981)
Supplement: Supplementary Notes S9 — (0.06 MB PDF) [file pone.0002981.s009.pdf]

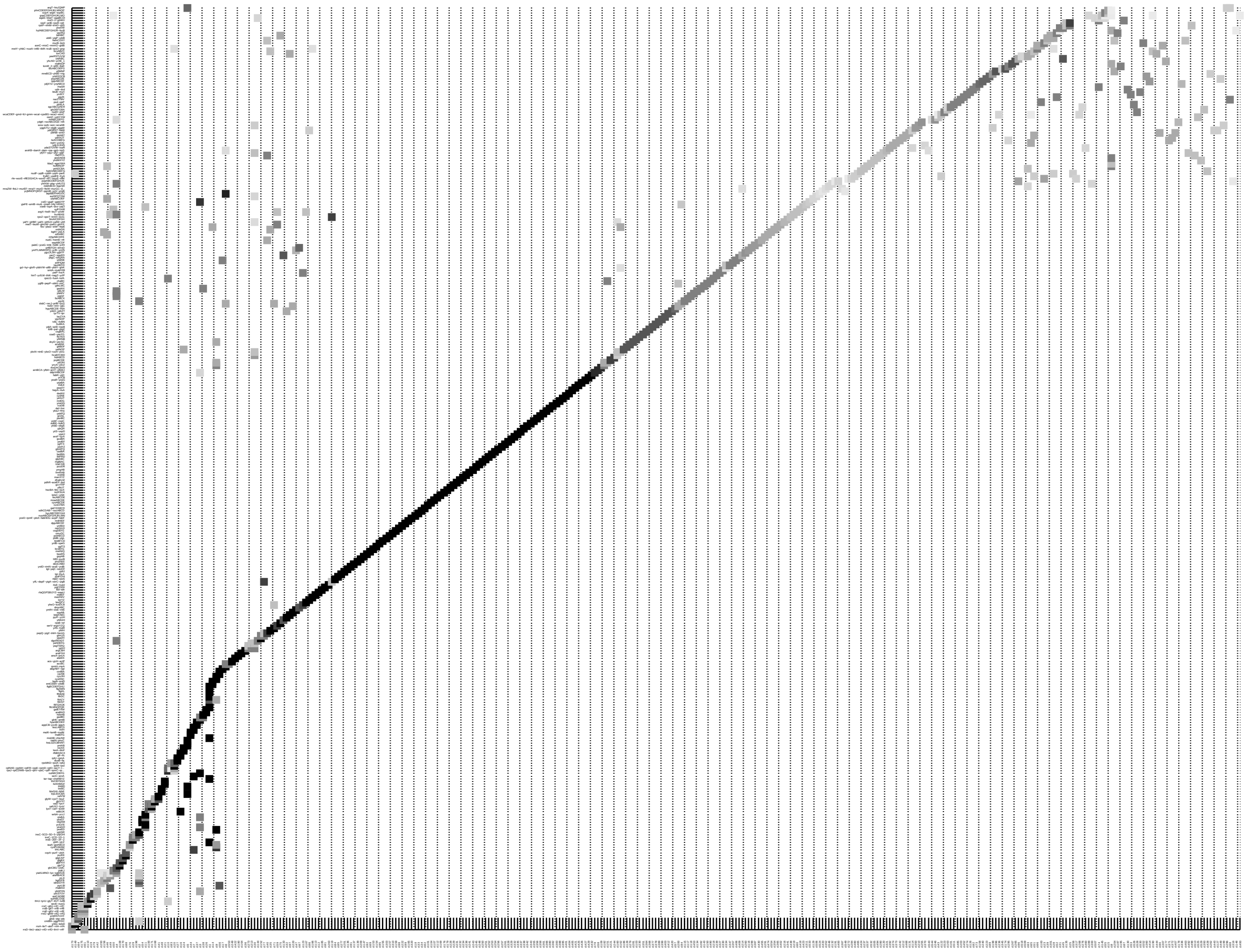

Figure S9: **Correspondence between expression clusters and TU physical network for *E.coli*.** Of the 556 expression clusters 365 intersect with 362 TU (that have nonempty intersection with the set of genes having a correlation $\geqslant 0.8$). The gray scale indicates the percentage of genes of the TU in the cluster (black is 100 %). The labels of this plot, TU names and cluster numbers, are available in the file "Labels for Fig. S9" downloadable at http://people.sissa.it/~altafini/papers/ZaSoBiAl07/. Statistics for this clustering are shown in Fig. S11.
