# Supplementary material for: Origin of Co-Expression Patterns in E.coli and S.cerevisiae Emerging from Reverse Engineering Algorithms
Source: PLoS One. 2008 Aug 20;3(8):e2981. doi: 10.1371/journal.pone.0002981 (PMC2500178; doi:10.1371/journal.pone.0002981)
Supplement: Supplementary Notes S10 — (1.80 MB PDF) [file pone.0002981.s010.pdf]

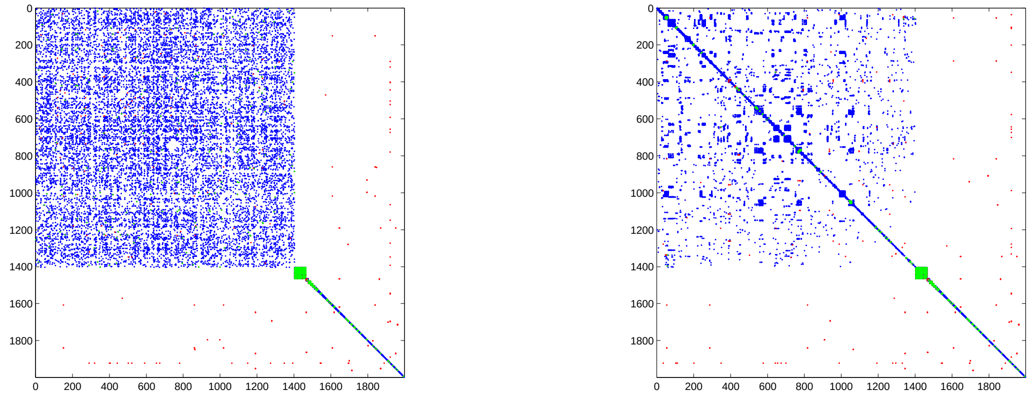

(a) (b)

Figure S10: **Clustering of the Pearson correlation matrix for *E.coli.*** The graph having as edges the 19238 gene pairs (blu dots) with Pearson correlation at least 0.8 and involving 1998 genes are first rearranged into 183 connected components (a). In red are shown the gene pairs involved in a protein complex that are not detected by the correlation with the 0.8 cut-off. In green are edges in a complex having correlation higher that 0.8. Notice how several diagonal blocks are completely green (meaning a connected component corresponding to a protein complex). In particular the larger green block corresponds to the flagellum cluster (see text). In (b) the large connected component is further clustered according to a hierarchical algorithm, see Supplementary Notes S1. With this clustering operation, a few more diagonal green blocks (corresponding to protein complexes) emerge.
