# Supplementary material for: Origin of Co-Expression Patterns in E.coli and S.cerevisiae Emerging from Reverse Engineering Algorithms
Source: PLoS One. 2008 Aug 20;3(8):e2981. doi: 10.1371/journal.pone.0002981 (PMC2500178; doi:10.1371/journal.pone.0002981)
Supplement: Supplementary Notes S12 — (0.04 MB PDF) [file pone.0002981.s012.pdf]

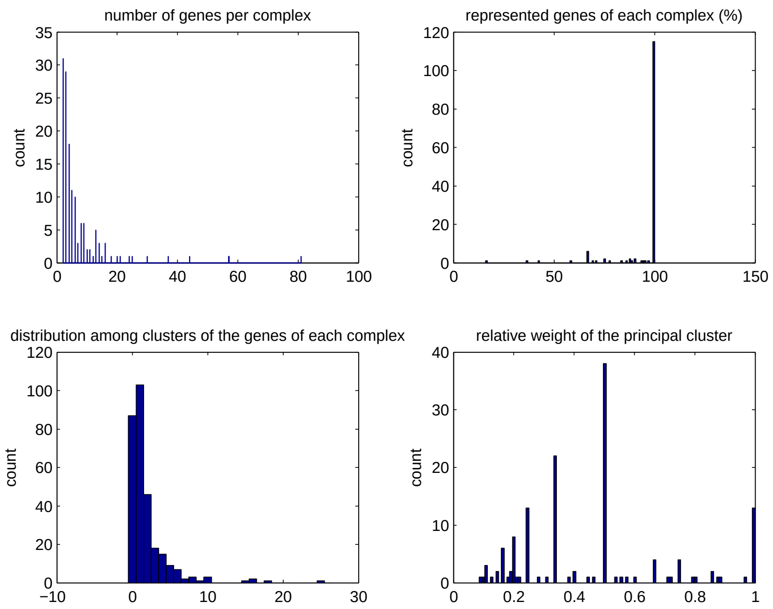

Figure S12: **Statistics for the clustered PC for *S.cerevisiae.*** Top left: histogram with the number of genes forming the PC; top right: histogram with the percentage of the PC-genes that are represented in the 1301 genes passing the correlation threshold in at least a case; bottom left: histogram of the number of clusters intersecting each PC; bottom right: histogram of the fraction of genes belonging to the "main" expression cluster for each PC.
