# Supplementary material for: Origin of Co-Expression Patterns in E.coli and S.cerevisiae Emerging from Reverse Engineering Algorithms
Source: PLoS One. 2008 Aug 20;3(8):e2981. doi: 10.1371/journal.pone.0002981 (PMC2500178; doi:10.1371/journal.pone.0002981)
Supplement: Supplementary Notes S13 — (14.39 MB PDF) [file pone.0002981.s013.pdf]

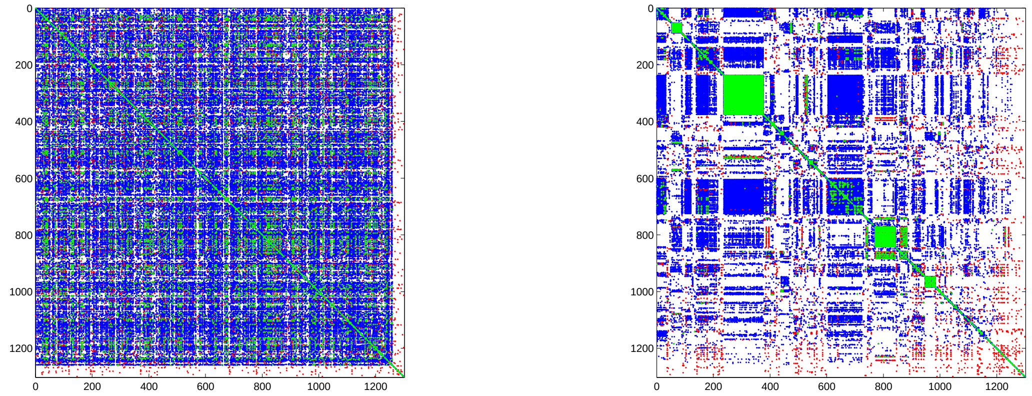

(a) (b)

Figure S13: **Clustering of the Pearson correlation matrix for *S.cerevisiae.*** The 131679 blu edges represent pairs of genes having correlation at least equal to 0.2 on both *S.cerevisiae* datasets. Overall they involve 1301 genes. Red dots represent gene pairs involved in protein complexes that do not correspond to edges in the correlation graph, green dots to correctly detected PC edges. Of the 16631 edges that correspond to gene pairs involved in protein complexes (restricted to the 1301 genes), 11417 are correctly identified. The blu graph is first explored looking for connected components (43). One of them is very large (1259 genes), see (a), and is further decomposed by means of hierarchical clustering, yielding a total of 299 clusters (including also the 42 remaining disconnected components), see (b). In doing so, large green diagonal blocks appear, corresponding to large protein complexes (partially) identified by the clustering. Notice how red dots (PC edges not corresponding to high correlations) are scattered throughout the plot.
