# Supplementary figures and images for: Origin of Co-Expression Patterns in E.coli and S.cerevisiae Emerging from Reverse Engineering Algorithms
Source: PLoS One. 2008 Aug 20;3(8):e2981. doi: 10.1371/journal.pone.0002981 (PMC2500178; doi:10.1371/journal.pone.0002981)

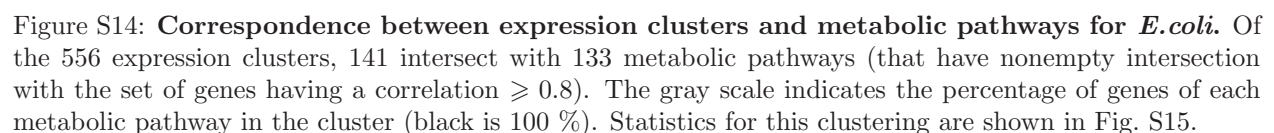

Supplement: Supplementary Notes S14 — (0.04 MB PDF) [file pone.0002981.s014.pdf]
