# Supplementary material for: Origin of Co-Expression Patterns in E.coli and S.cerevisiae Emerging from Reverse Engineering Algorithms
Source: PLoS One. 2008 Aug 20;3(8):e2981. doi: 10.1371/journal.pone.0002981 (PMC2500178; doi:10.1371/journal.pone.0002981)
Supplement: Supplementary Notes S15 — (0.04 MB PDF) [file pone.0002981.s015.pdf]

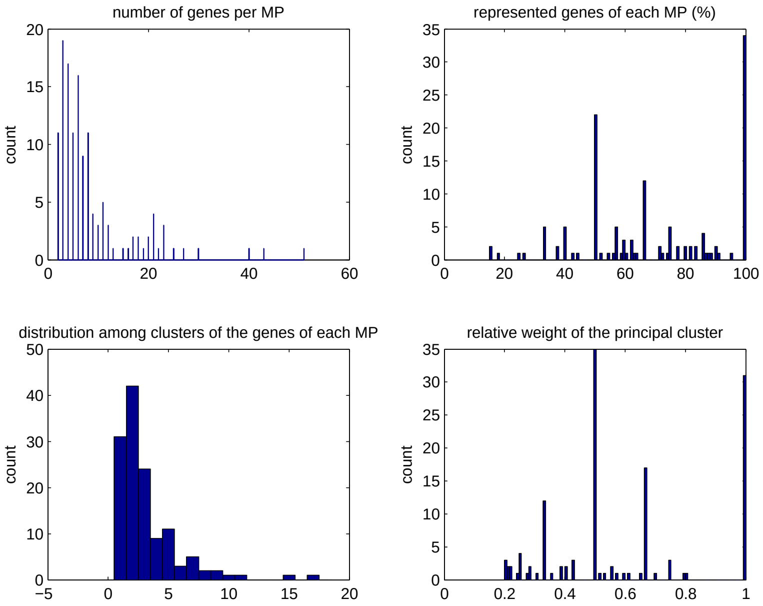

Figure S15: **Statistics for the clustered MP for *E.coli.*** Top left: histogram with the number of genes forming the MP; top right: histogram with the percentage of the enzymatic genes that are represented in the 1998 genes passing the correlation threshold; bottom left: histogram of the number of clusters intersecting the MP; bottom right: histogram of the fraction of genes belonging to the "main" expression cluster for each MP.
